# Overexpression of *SlGRAS40* in Tomato Enhances Tolerance to Abiotic Stresses and Influences Auxin and Gibberellin Signaling

**DOI:** 10.3389/fpls.2017.01659

**Published:** 2017-09-26

**Authors:** Yudong Liu, Wei Huang, Zhiqiang Xian, Nan Hu, Dongbo Lin, Hua Ren, Jingxuan Chen, Deding Su, Zhengguo Li

**Affiliations:** School of Life Sciences, Chongqing University, Chongqing, China

**Keywords:** abiotic stresses, auxin, gibberellin, GRAS transcription factor, *SlGRAS40*, tomato (*Solanum lycopersicum*)

## Abstract

Abiotic stresses are major environmental factors that inhibit plant growth and development impacting crop productivity. GRAS transcription factors play critical and diverse roles in plant development and abiotic stress. In this study, *SlGRAS40*, a member of the tomato (*Solanum lycopersicum*) GRAS family, was functionally characterized. In wild-type (WT) tomato, *SlGRAS40* was upregulated by abiotic stress induced by treatment with D-mannitol, NaCl, or H_2_O_2_. Transgenic tomato plants overexpressing *SlGRAS40* (*SlGRAS40*-OE) were more tolerant of drought and salt stress than WT. *SlGRAS40*-OE plants displayed pleiotropic phenotypes reminiscent of those resulting from altered auxin and/or gibberellin signaling. A comparison of WT and *SlGRAS40*-OE transcriptomes showed that the expression of a large number of genes involved in hormone signaling and stress responses were modified. Our study of *SlGRAS40* protein provides evidence of how another GRAS plays roles in resisting abiotic stress and regulating auxin and gibberellin signaling during vegetative and reproductive growth in tomato.

## Introduction

Tomato (*Solanum lycopersicum*) is susceptible to a wide range of environmental stresses. Drought and high salinity are major stress factors leading to detrimental effects, such as inhibition of seed germination and plant growth and decreased fruit productivity. Under drought and salt stress, a series of stress related genes are induced in plants, largely regulated by a range of transcription factors (Hirayama and Shinozaki, 2010). To date, transcription factors from various plant species have been reported to be involved in stress responses (Singh et al., 2002), such as NAC, Dof, ERF, WRKY, bZIP, and MYC/MYB.

Mounting evidence shows that *GRAS* (named after GAI, RGA, SCR) genes are highly inducible by different abiotic stresses (Czikkel and Maxwell, 2007; Lee et al., 2008). GRAS family proteins are plant-specific transcription factors that play critical roles in plant development and signal transduction pathways, including gametogenesis (Morohashi et al., 2003), phytochrome signaling (Torres-Galea et al., 2006), lateral shoot formation (Li et al., 2003), gibberellin biosynthesis and signaling (Silverstone et al., 1998; Lee et al., 2002), and auxin signaling (Gao et al., 2004; Sanchez et al., 2007). However, some experimental evidence points to certain GRAS proteins having roles in resisting biotic or abiotic stress. Tomato *SlGRAS6* silenced plants showed increased resistance to disease (Mayrose et al., 2006), overexpression of *OsGRAS23* improved drought and oxidative stress tolerance in rice (Kai et al., 2015), and ectopic expression of *Populus PeSCL7* gene in *Arabidopsis* improved drought and salt tolerance (Ma et al., 2010). Overexpression of *VaPAT1*, a GRAS transcription factor from *Vitis amurensis*, in transgenic *Arabidopsis* conferred cold, drought and high salinity tolerance (Yuan et al., 2016). Identifying the roles of other GRAS genes in crops, like tomato, will help elucidate the mechanisms regulating stress tolerance and potentially facilitate the breeding of resistant varieties.

In planta, auxin and gibberellin (GA) are inextricably linked with both developmental processes and abiotic stress responses through the action of GRAS proteins from the SCARECROW-like (SCL) and DELLA groups. AtSCL3 acts as an integrator of DELLAs and SHR-SCR, a complex in which two types of GRAS interact, to mediate GA-promoted cell elongation in the root endodermis (Heo et al., 2011; Zhang Z. L. et al., 2011). The DELLA mutants *AtGAI* (Peng et al., 1997), *AtRGA* (Silverstone et al., 1998), and *AtRGL1-3* (Lee et al., 2002) all exhibit gibberellin insensitivity. It has been shown that decreased GA content enhances tolerance to drought, whereas increased GA content reduces it (Colebrook et al., 2014). DELLA proteins, the core components of GA signaling, may therefore restrain growth and enhance stress tolerance through a common mechanism (Achard et al., 2008). PrSCL1 (*Pinus radiata* SCL1) in pine and CsSCL1 (*Castanea sativa* SCL1) in chestnut regulate adventitious root formation by regulating auxin signaling (Sanchez et al., 2007). The SHR-SCR complex combined with auxin influx carriers LAX3 and AUX1 has a synergetic effect on primary/lateral root development in *Arabidopsis* (Della et al., 2015). Auxin directly or indirectly modulates the expression of several stress-responsive genes, and several auxin-responsive genes are regulated by abiotic stresses (Jain and Khurana, 2009). Besides, auxin can modulate ROS homeostasis indirectly by affecting the stability of DELLA proteins or directly by inducing ROS detoxification enzymes, suggesting that auxin and GA might cooperate with each other in response to stress conditions (Fu and Harberd, 2003; Paponov et al., 2008).

There are 53 members of the GRAS family in tomato (Huang et al., 2015). In our previous study, we demonstrated that *SlGRAS24* participates in a series of developmental processes in tomato by modulating auxin and gibberellin crosstalk (Huang et al., 2017). Here, we found that overexpression of *SlGRAS40* (Solyc08g078800) conferred pleiotropic phenotypes and enhanced salt and drought tolerance. By analyzing hormone responsiveness and gene expression we show that altered auxin and gibberellin signaling are likely to be responsible for the defective growth of *SlGRAS40*-OE.

## Materials and methods

### Plant materials and growth conditions

Tomato plants (*Solanum lycopersicum* cv. Micro-Tom) were grown in the greenhouse in controlled conditions with 18 h light (25°C)/6 h dark (18°C) cycles and 60% relative humidity. For gene expression analysis the following samples were collected from at least six plants: roots, stems, and leaves from 1-month-old WT plants; buds, flowers, petals, sepals, and stamens at anthesis; ovaries at −2, 0, and 4 dpa; and fruits at 9 dpa, 20 dpa, mature green, breaker, breaker plus 4 day, and breaker plus 7 day stages. Samples from different plants were mixed and immediately frozen in liquid nitrogen.

### Plasmid construction and genetic transformation

The open reading frame of *SlGRAS40* without the stop codon was amplified from the full length tomato leaf cDNA and cloned into the expression vector under the CaMV 35S promoter. The expression vector was transformed into *Agrobacterium tumefaciens* strain GV3101, which was used to transform WT tomato plants following standard methods (Fillatti et al., 1987).

### Gene expression analysis

Total RNA was extracted using an RNAeasy kit (QIAGEN, Germany) and first-strand cDNA was synthesized with PrimeScript™ RT reagent Kit with gDNA Eraser (Perfect Real Time) (TAKARA, Japan). Quantitative real-time PCR was performed according to the instructions provided for the Bio-Rad CFX system (Bio-Rad, USA), using SYBR® Premix Ex Taq™ (Tli RNaseH Plus) (TAKARA, Japan). Relative fold differences were calculated by 2^−ΔΔCt^ method based on the comparative Ct method. For each sample, three independent biological replicates were used (each with three technical replicates). And all the primers used for qRT-PCR were listed in Supplementary Table S1.

### Histological analysis

Stems of 1-month-old WT and OE L3 plants were cut and soaked in FAA solution (50% (v/v) ethanol, 5% (v/v) glacial acetic acid, 5% (v/v) methanal) for 24 h. Samples were dehydrated in an ethanol gradient then embedded in paraffin. Sections were cut and stained with 0.05% toluidine blue. An Olympus BX-URA2 (Japan) microscopy was used for observations.

### Abiotic stress treatments

Leaves of 1-month-old WT plants were sprayed with 200 mM NaCl, 100 mM D-mannitol or 100 mM hydrogen peroxide for salt, osmotic, and oxidative treatments, respectively. Control plants were untreated. The leaves were harvested after 1, 3, 6, 12, and 24 h. For each sample, leaves from six plants were mixed and all treatments were performed three independent times. All samples were frozen in liquid nitrogen and stored at −80°C.

For drought tolerance assay, every plant was planted in an independent square basin (same basins were used), each 12 plants of WT and *SlGRAS40*-OE L2, L3, L4 were placed in a big pot, and watered into the big pot twice a week to make sure the soil water in every basin was uniform, and all the plants were grown under some illumination and temperature conditions. After 1 month, WT and *SlGRAS40*-OE plants in the big pot were average divided into two groups, one group as control was watered normally, and the other group as drought treatment was deprived of water for up to 17 days. Similarly, the group as salt treatment was watered with 200 mM NaCl every 48 h (200 ml per plant) for up to 23 days. During the treatment process, all the plants in each groups were grown under same illumination and temperature conditions, and three independent treatments were performed. Total chlorophyll content and relative water content (RWC) (Pan et al., 2012) were measured during treatment. After drought or salt treatment, leaf samples at the same developmental stage were harvested, frozen in liquid nitrogen immediately, and stored at −80°C.

For osmotic and salt tolerance experiments, seeds of WT and *SlGRAS40*-OE L2, L3, and L4 were sterilized and sown on ½ × MS alone or ½ × MS containing 150 mM D-mannitol or 75 mM NaCl (Huang et al., 2016). Seeds were incubated in a controlled growth chamber with 18 h light (25°C)/6 h dark (18°C) cycles. The germination rate was counted after 7 days, and the lengths of primary roots and hypocotyls were measured after 2 weeks.

### Stoma morphology and stomatal conductance assay

An imprint method was used for the assay. Colorless nail polish was applied to the underside of leaves of WT and OE L3 plants. After 5 min, the nail polish was carefully torn off and the imprint viewed with an Olympus BX-URA2 (Japan) microscope. The length and width of stoma were measured with ImageJ software. Stomatal conductance was estimated as the stoma length/width.

### Analysis of antioxidant enzyme activities and MDA, proline, and soluble sugar content

After drought and salt treatment, leaves at the same developmental stage were harvested for measuring enzyme activities and Malondialdehyde (MDA), proline and soluble sugar content. MDA content was measured according to the method by Heath and Packer (1968), proline content with the method described by Bates et al. (1973), and soluble sugar content by the method described by Fukao et al. (2006). The activity of superoxide dismutase (SOD), catalase (CAT) and peroxidase (POD) were determined according to the methods described by Mittova et al. (2000) and by Morohashi (Mittova et al., 2000; Morohashi, 2002). H_2_O_2_ content and the superoxide anion radical (${\text{O}}_{2}^{-}$) content were determined according to the instructions provided in kits available from Jiancheng Bioengineering Company (Nanjing, China).

### Pollen germination and pollen tube growth assays

Pollen from WT and OE L3 plants at flowering time was tested in pollen germination assay. A 30 μl drop of medium (20 mM MES buffer, pH6.0, 2% sucrose, 15% PEG4000, 1 mM KNO_3_, 3 mM Ca(NO_3_)_2_·4H_2_O, 0.8 mM MgSO_4_·7H_2_O, 1.6 mM H_3_BO_3_) was placed on a glass slide in a moist petri dish. A flower undergoing anthesis was shaken to let pollen fall onto the medium. The petri dish was sealed and kept in the dark at 25°C for 5–6 h. Observations were made with an Olympus BX-URA2 microscope (Japan). For pollen tube growth assays, 1-dpa ovaries were soaked in buffer (95% ethanol-glacial acetic acid, v/v, 3:1) for 24 h, then transferred to 8 M NaOH for 2 days. Samples were washed in water three times, and stained with 0.05% aniline blue for 4 h in the dark. An Olympus BX-URA2 microscope (Japan) with ultraviolet light excitation was used for observations of pollen tubes.

### Hormone treatment for plant development analysis

Ten-day-old WT and *SlGRAS40*-OE L3 plants were sprayed with 20 μM GA_3_ every 3 days for 4 weeks. Control plants were sprayed with water. Plant height was measured every week and flowering time was recorded. The auxin dose-response experiment was performed with 8-mm long hypocotyl segments excised just below the cotyledon nodes from 7-day-old WT and *SlGRAS40*-OE L3 seedlings. Hypocotyl segments were floated onto sucrose/MES buffer [1% (w/v) sucrose, 5 mM MES/KOH, pH6.0] and pre-incubated for 1–2 h. The hypocotyl segments were then randomly distributed to fresh buffer solutions with or without NAA and measured after 23 h of incubation with gentle agitation at room temperature (Wang et al., 2005).

The seeds of WT and *SlGRAS40*-OE L2 and L3 were sterilized and soaked in sterilized water with gentle agitation at room temperature, then seeds were transferred to ½ × MS medium with 1 μM IAA and/or 50 μM GA_3_, or on ½ × MS medium without hormone for controls. The seedlings were grown in a growth chamber with controlled conditions, 18 h light (25°C)/6 h dark (18°C) cycles. After 15 days, the number of lateral roots, and the lengths of primary roots and hypocotyls were measured. Every treatment was done on at least 10 plants or 30 seeds and all the treatments were performed three independent times.

### Hormone treatment for gene expression analysis

Fifteen-day-old WT seedlings were soaked in liquid ½ × MS medium containing 20 μM IAA or 20 μM GA_3_ for 0, 1, 3, 6, 12, or 24 h, then whole seedlings were frozen in liquid nitrogen and stored at −80°C.

Fifteen-day-old WT and OE L3 seedlings were soaked in liquid ½ × MS medium containing 20 μM IAA or 20 μM GA_3_ for 3 h, then whole seedlings were frozen in liquid nitrogen and stored at −80°C. Seedlings were soaked in liquid ½ × MS medium without hormones as controls. Each treatment was performed three independent times.

GA_3_ (2000 ng per ovary, Sigma, USA) or 2,4-D (200 ng per ovary, Sigma, USA) was applied to emasculated ovaries on the day equivalent to anthesis (0 dpa) in 10 μl of 5% ethanol and 0.1% Tween 80 (Sigma, USA) solution. Ovaries were treated with the same volume of solution without hormones as control. Eight ovaries (from four plants) were used per treatment, and each treatment was performed three independent times. The samples were frozen in liquid nitrogen and stored at −80°C before RNA extraction and gene expression analysis. Flower emasculation was carried out 2 days before anthesis (−2 dpa) to prevent self-pollination (Serrani et al., 2007a).

### RNA sequencing

Total RNA (RNeasy Plant Mini Kit, Qiagen, USA) was extracted from shoot apical meristems from 1-month-old WT and *SlGRAS40*-OE L3 plants, two individuals per sample. A cDNA library was constructed for sequencing on the IlluminaHiSeq2000™ system (BGI Inc.). *Bowtie2* was used to map clean reads to the reference genome of *S. lycopersicum* in the Tomato Sol Genomic Network database (http://solgenomics.net/), and the homogenized data used to calculate gene expression levels with *RSEM*. Differentially expressed genes (DEGs) were detected with NOIseq with the following parameters: fold change ≥ 2.00 and probability ≥ 0.8. Gene ontology (GO) functional enrichment and Kyoto Encyclopedia of Genes and Genomes (KEGG) pathway analysis were performed between WT and *SlGRAS40*-OE samples.

### Statistical analysis

All the experiments included three independent repeats, and significant differences were determined by the Student's *t*-test at significance levels of *P* < 0.05 (^*^) and *P* < 0.01 (^**^).

## Results

### Phenotypic characterization of transgenic plants overexpressing *SlGRAS40*

**Figure 1:**
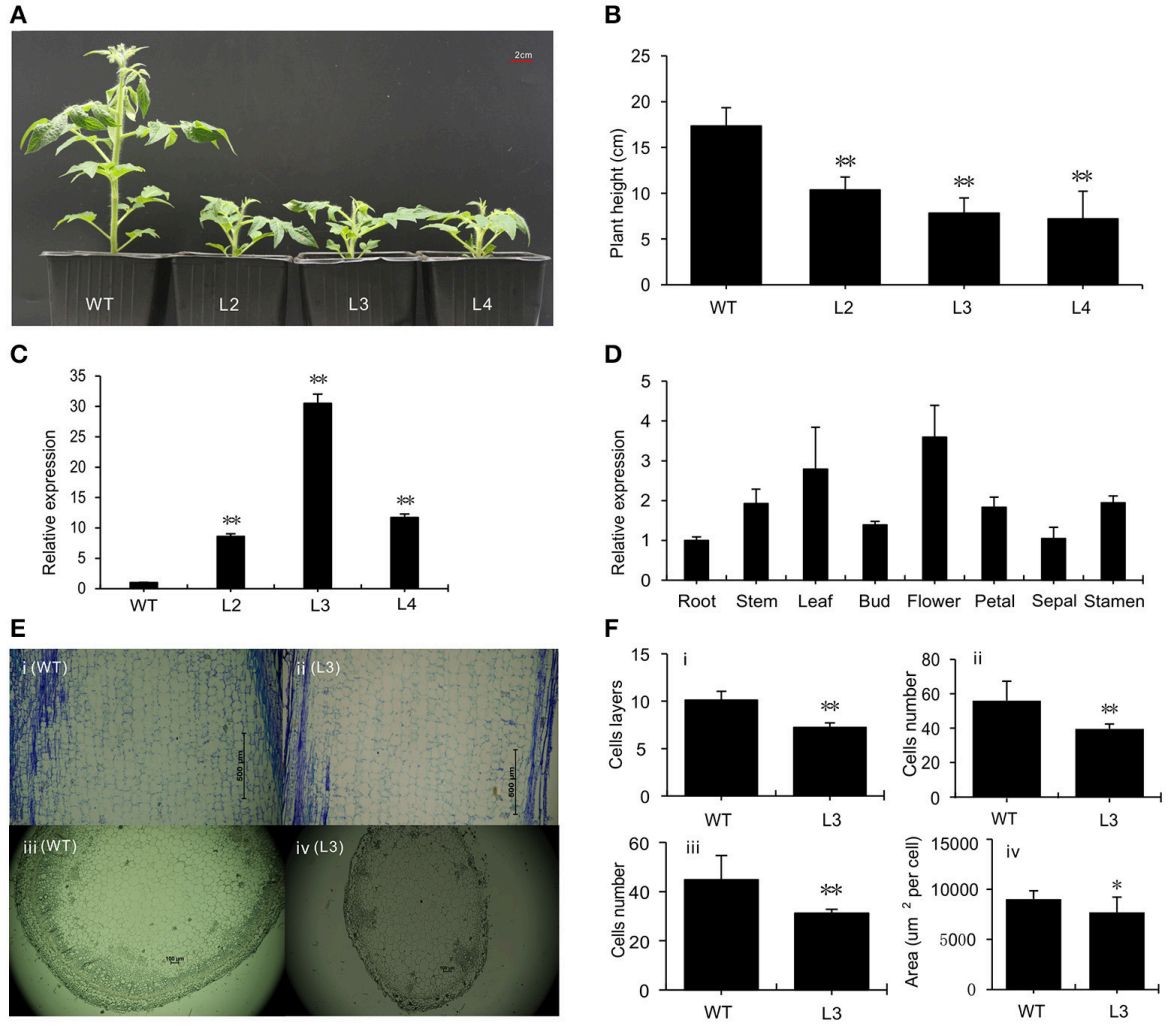
Phenotypic characterization of wild-type and transgenic plants. **(A)** One-month-old plants of WT and *SlGRAS40*-overexpressing lines. L2, L3, L4, three independent *SlGRAS40*-overexpressing lines. **(B)** Height of plants shown in **(A)**. Error bars show the standard error between three biological replicates (*n* = 3) with more than 10 plants for each replicate performed. **(C)** Expression levels of *SlGRAS40* in plants of WT and *SlGRAS40*-OE lines. Expression data were normalized with *SlGRAS40* expression in WT as 1. Error bars show the standard error between three biological replicates (*n* = 3). **(D)** Tissue profiling analysis of *SlGRAS40* in different organs of wild-type tomato. Expression data were normalized with *SlGRAS40* expression in root set as 1. **(E)** Histological analysis of 1-month-old stems from WT (i, iii) and *SlGRAS40*-OE L3 (ii, iv) shown in longitudinal section (i, ii) and transverse section (iii, iv). **(F)** Analysis of histological data from **(E)**. (i) and (ii) are data from longitudinal sections; (iii) and (iv) are data from transverse sections. All data are measurements under 40× microscopic field. Asterisks indicate significant differences using Student's *t*-test (^*^P < 0.05, ^**^P < 0.01).

We generated transgenic tomato plants with the *SlGRAS40* cDNA under the control of the CaMV 35S promoter by *Agrobacterium tumefaciens*-mediated transformation. Three independent lines L2, L3, L4 were obtained overexpressing the gene 8.6-fold, 30.5-fold, and 11.7-fold, respectively (Figures 1A,C). *SlGRAS40* overexpressing plants exhibited pleiotropic phenotypes including dwarfism (Figure 1B), delayed flowering time, decreased fruit-set ratio, and arrested fruit and seed development. More details of the phenotypes are shown in Supplementary Table S2. *SlGRAS40* was expressed in all WT tissues tested, and expressed at much higher levels in leaves and flowers undergoing anthesis compared to roots (Figure 1D).

The sixth node stems of 1-month-old WT and *SlGRAS40*-OE L3 plants were used for histological analysis (Figure 1E). In longitudinal sections, there were fewer cell layers and fewer cells in OE plants compared with WT, and the average area of OE cells was bigger than WT cells. In transverse section, OE plants had fewer and smaller cells compared to WT (Figure 1F). The difference in cellular makeup explains why *SlGRAS40*-OE stems are thinner and shorter than WT stems.

### Overexpression of *SlGRAS40* enhances tolerance to drought and salt stress

**Figure 2:**
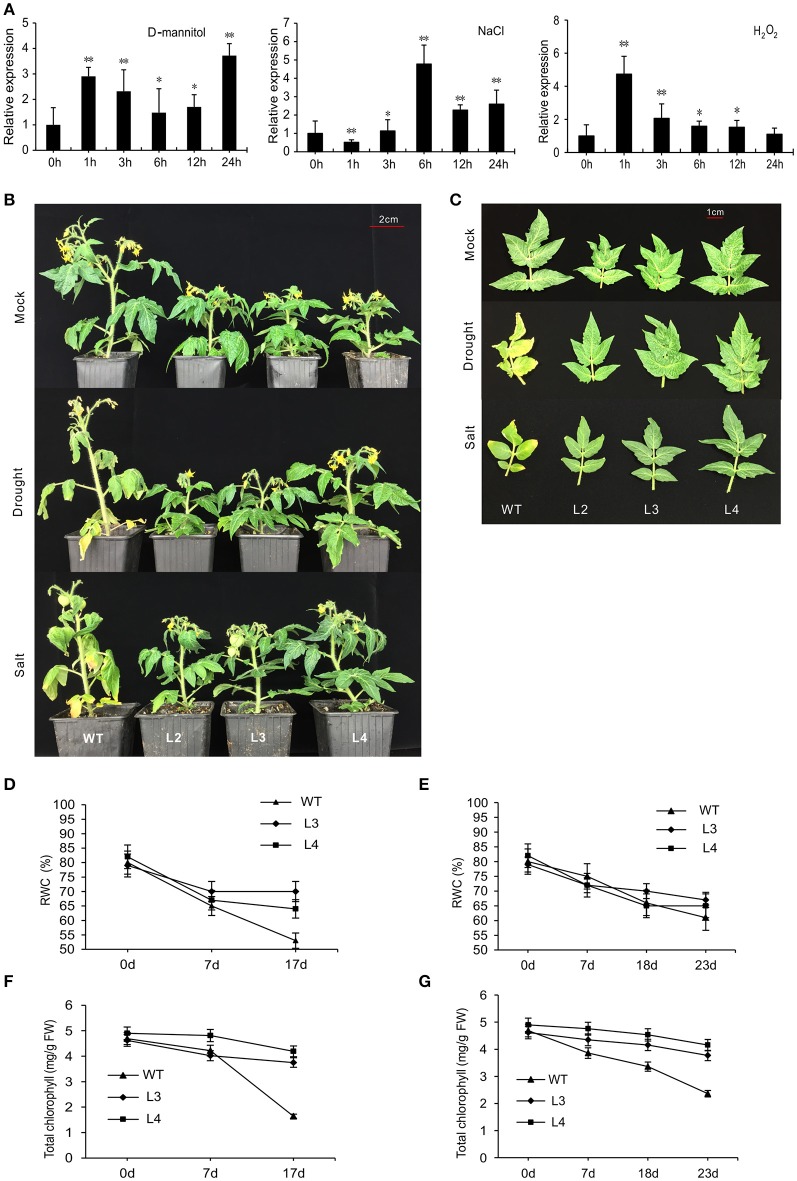
Overexpression of *SlGRAS40* enhances tolerance to drought and salt stress. **(A)** Quantitative RT-PCR analysis of *SlGRAS40* mRNA from leaves of 1-month-old WT plants sprayed with 100 mM D-mannitol, 200 mM NaCl ,or 100 mM H_2_O_2_. Expression data was normalized with expression of *SlGRAS40* in treated plants at 0 h set as 1. Asterisks indicate significant differences using Student's *t*-test (^*^P < 0.05, ^**^P < 0.01). **(B)** Photographs of representative plants after 17 days of drought treatment or 23 days of salt treatment compared to control plants. **(C)** Phenotypes of the fifth leaves of plants shown in **(B)**. RWC **(D,E)** and total chlorophyll content **(F,G)** were measured after drought **(D,F)** and salt **(E,G)** stress treatment. Error bars show the standard error of data from three replicates.

WT plants were treated with D-mannitol, NaCl, or H_2_O_2_ to produce the effects of osmotic, saline or oxidative stress, respectively. *SlGRAS40* was up-regulated in response to 100 mM D-mannitol and to 100 mM H_2_O_2_, but in response to 200 mM NaCl *SlGRAS40* was first down-regulated then up-regulated (Figure 2A). *SlGRAS40* may therefore be involved in abiotic and oxidative stress responses in tomato.

**Figure 3:**
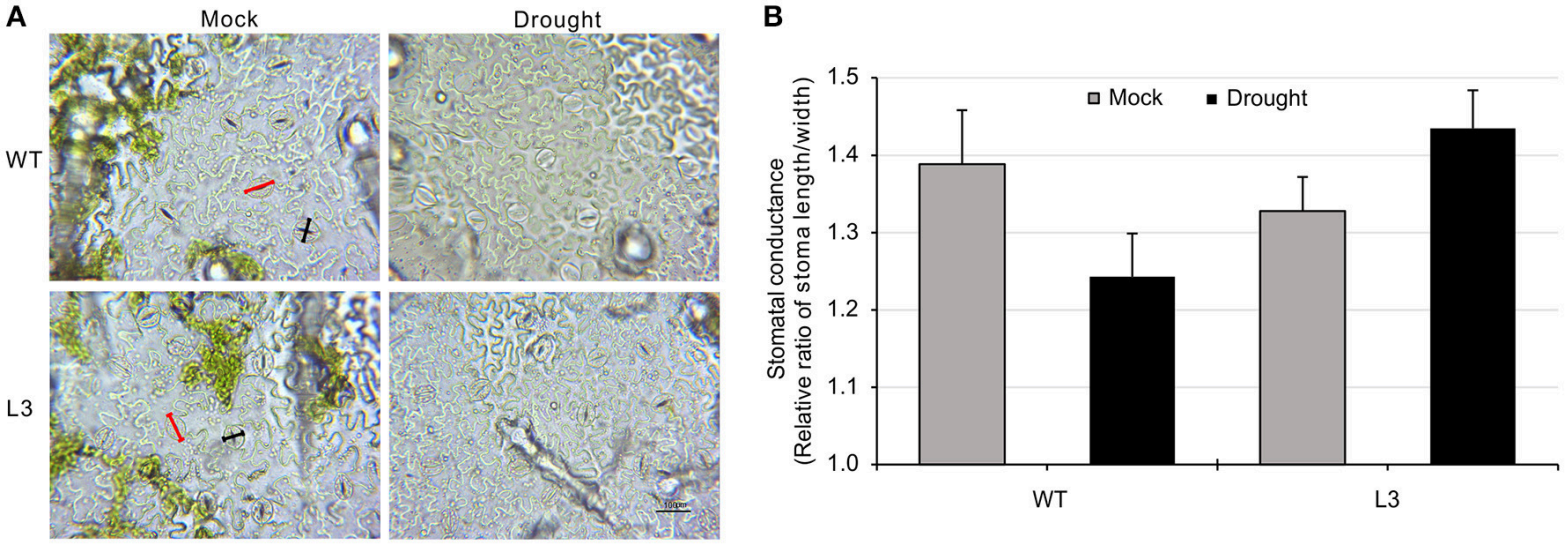
Overexpression of *SlGRAS40* reduces stoma opening under drought stress. **(A)** Stoma morphology of WT and *SlGRAS40*-OE L3 leaves under mock and drought conditions. Red indicates pore length, black indicates pore width. **(B)** Stomatal conductance of WT and *SlGRAS40*-OE L3 leaves under mock and drought conditions. Stomatal conductance = pore length/pore width. Error bars show the standard error between three biological replicates (*n* = 3) with more than five leaves per replicate.

To evaluate the role of *SlGRAS40* in tolerance to drought and salt, *SlGRAS40*-OE L2, L3, L4 and WT plants were deprived of water for up to 17 days as a drought stress treatment, or watered with 200 mM NaCl solution every 48 h for up to 23 days as a salt stress treatment. Under saline or drought stresses, all OE plants grew better than WT (Figure 2B). Under drought stress, desiccation symptoms, such as wilting of lower leaves were readily observed in WT, whereas OE plants exhibited only slight damage (Figure 2C). Under salt stress, WT plants mostly exhibited increased chlorosis and necrosis after 23 days, but there was no obvious damage in OE plants (Figure 2C). Relative water content (RWC) and total chlorophyll content both declined in WT and OE plants during stress treatments, but levels were much higher in OE than in WT (Figures 2D–G). After drought stress, the stomatal conductance of WT had decreased (Figures 3A,B), indicating that stomatal pores were wider open. The effect would be to increase transpiration leading to lower RWC (Figure 2D). On the contrary, after drought stress the stomatal conductance of *SlGRAS40*-OE L3 leaves increased and was significantly higher than that of WT leaves (Figure 3B). This indicated that under drought stress the stomatal pores of *SlGRAS40*-OE leaves were narrowed so more water was retained (Figure 2D).

### Antioxidant status of *SlGRAS40*-OE plants under drought and salt stress

**Figure 4:**
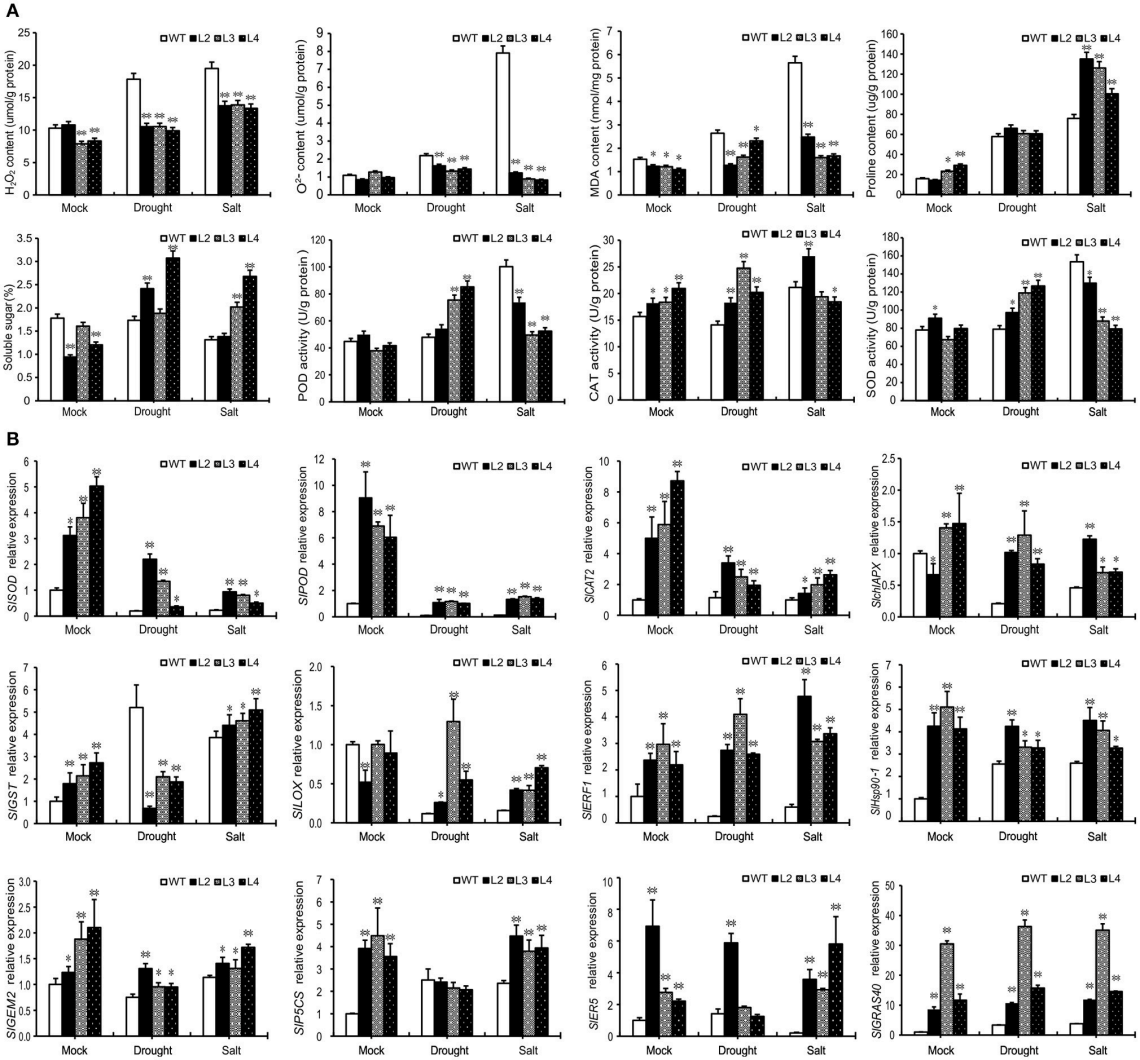
Overexpression of *SlGRAS40* elevates ROS scavenging ability. **(A)** H_2_O_2_, O^2−^, MDA, proline, and soluble sugar contents and POD, CAT, and SOD activities in leaves of WT and *SlGRAS40*-OE plants under normal and stress conditions. Leaf samples were from the plants shown in Figure 2B. **(B)** Transcript levels of stress related genes in wild-type and transgenic plants under normal and stress conditions. Leaf samples from the plants shown in Figure 2B were used for RNA extraction. Error bars show the standard error between three replicates performed. Asterisks indicate significant differences using Student's *t*-test (^*^P < 0.05, ^**^P < 0.01).

Abiotic stresses induce oxidative stress in plants which leads to the accumulation of abundant ROS. We investigated whether H_2_O_2_ and ${\text{O}}_{2}^{-}$ accumulate in WT and OE L2, L3, L4 plants after drought or salt treatment. Results show that drought and salt stress induced more H_2_O_2_ and ${\text{O}}_{2}^{-}$ to accumulate in WT plants, but there was no obvious change in OE plants (Figure 4A). MDA, proline, and soluble sugar contents were measured in treated and control plants (Figure 4A), as accumulation of these compounds is characteristic of physiological stress. MDA content increased significantly in WT plants under drought and salt stress, and slightly increased in OE plants but not as much as in WT. The proline content of both WT and OE plants increased after drought and salt stress, but more proline accumulated in OE plants under salt stress. There was an obvious decrease in soluble sugar in WT plants after salt treatment, whereas OE plants accumulated more soluble sugar after drought and salt stress.

The activities of three antioxidant enzymes, POD, SOD, and CAT, were measured in this study. There were no significant differences in either POD or SOD activities in WT and OE plants under normal conditions. Under drought stress both POD and SOD activities were up-regulated in OE plants, but not in WT plants. By comparison, these two activities were significantly up-regulated in WT plants after salt treatment (Figure 4A). Salt-stressed OE had lower levels of POD and SOD than WT in the same conditions. CAT activity was higher in OE than in WT plants under both control and drought stress conditions, but after salt treatment the CAT activity was up-regulated in WT but not in OE (Figure 4A). These data indicate that overexpression of *SlGRAS40* can enhance ROS scavenging ability under salt and drought stress.

### Expression analysis of stress-related genes in *SlGRAS40*-OE and WT plants under drought and salt stress

To investigate the molecular mechanisms underlying *SlGRAS40*-enhanced tolerance to drought and salt stress, expression of plant stress response biomarkers was checked by quantitative reverse transcription (qRT)-PCR (Figure 4B). The transcript levels of several genes involved in ROS generation and scavenging, including ascorbate peroxidase (*APX*), *CAT, SOD, POD*, lipoxygenase (*LOX*), and glutathione S-transferase (*GST*), were measured in WT and transgenic plants under both normal and stress conditions. The transcript abundance of all these genes, except *SlGST*, decreased in WT and OE plants after stress treatment, but the expression levels in OE plants were higher than those in WT in both control and stress conditions (Figure 4B). *SlGST* was up-regulated in WT after drought or salt stress, and up-regulated in OE plants after salt stress, but after drought stress *SlGST* expression level was lower in OE than in WT. A key proline synthetase gene *SlP5CS* was up-regulated after stress treatment in WT plants, while OE plants had higher levels than WT under control and salt conditions (Figure 4B). An ascorbic acid synthetase (*SlGME2*), an ethylene-responsive LEA protein (*SlER5*), an ethylene-responsive factor (*SlERF1*) and a heat shock protein (*SlHsp90-1*) all have higher expression levels in OE plants than in WT after drought and salt stress (Figure 4B). These results indicated that *SlGRAS40* may be involved in stress signaling pathways by modulating these genes in tomato.

### Overexpression of *SlGRAS40* improves seed germination rate under osmotic and salt stress

**Figure 5:**
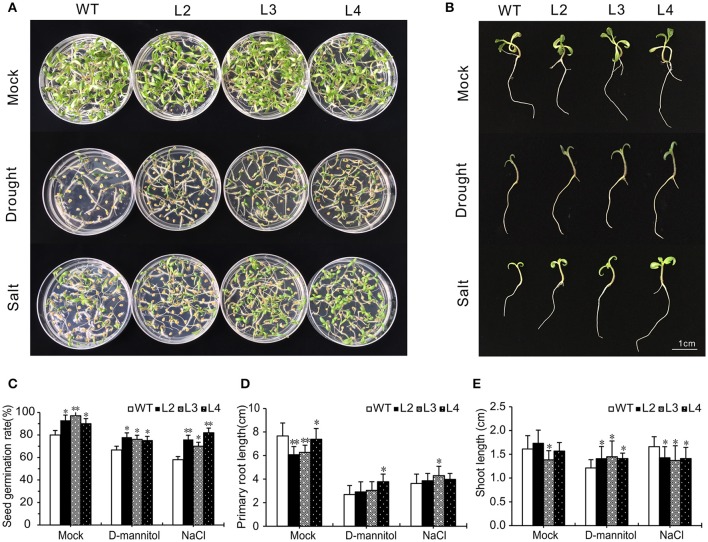
Comparative analysis of WT and *SlGRAS40*-OE lines under drought and salt stress. **(A, B)** Seed germination of WT and *SlGRAS40*-OE lines after 2 weeks in medium containing 150 mM D-mannitol or 75 mM NaCl. **(C)** Germination rate of WT and *SlGRAS40*-OE lines under control and drought or salt treatments. **(D)** Primary root length of WT and *SlGRAS40*-OE seedlings under control and drought or salt treatments. **(E)** Shoot length of WT and *SlGRAS40*-OE seedlings under control and drought or salt treatments. Error bars show the standard errors between three replicates. Asterisks indicate significant differences using Student's *t*-test (^*^P < 0.05, ^**^P < 0.01).

The osmotic and salt tolerance of seed germination was tested on seeds harvested from WT and OE L2, L3, L4 plants (Figures 5A,B). The germination rate of both genotypes declined under 150 mM D-mannitol and 75 mM NaCl, respectively (Figure 5C). The germination rate of OE seeds was significantly higher than that of WT seeds under either stress condition, on average 76.3 vs. 66.7% after osmotic treatment and 76 vs. 58% after salt treatment.

Root elongation was significantly delayed under osmotic and salt stress, but OE root length was longer than WT root length under the corresponding stress treatments (Figure 5D). OE hypocotyls were longer than WT after D-mannitol treatment, but shorter than WT under salt stress (Figure 5E). These results showed that *SlGRAS40*-OE seeds and seedlings resist drought and salt stress better than WT.

### Overexpression of *SlGRAS40* alters responsiveness to IAA and GA_3_

**Figure 6:**
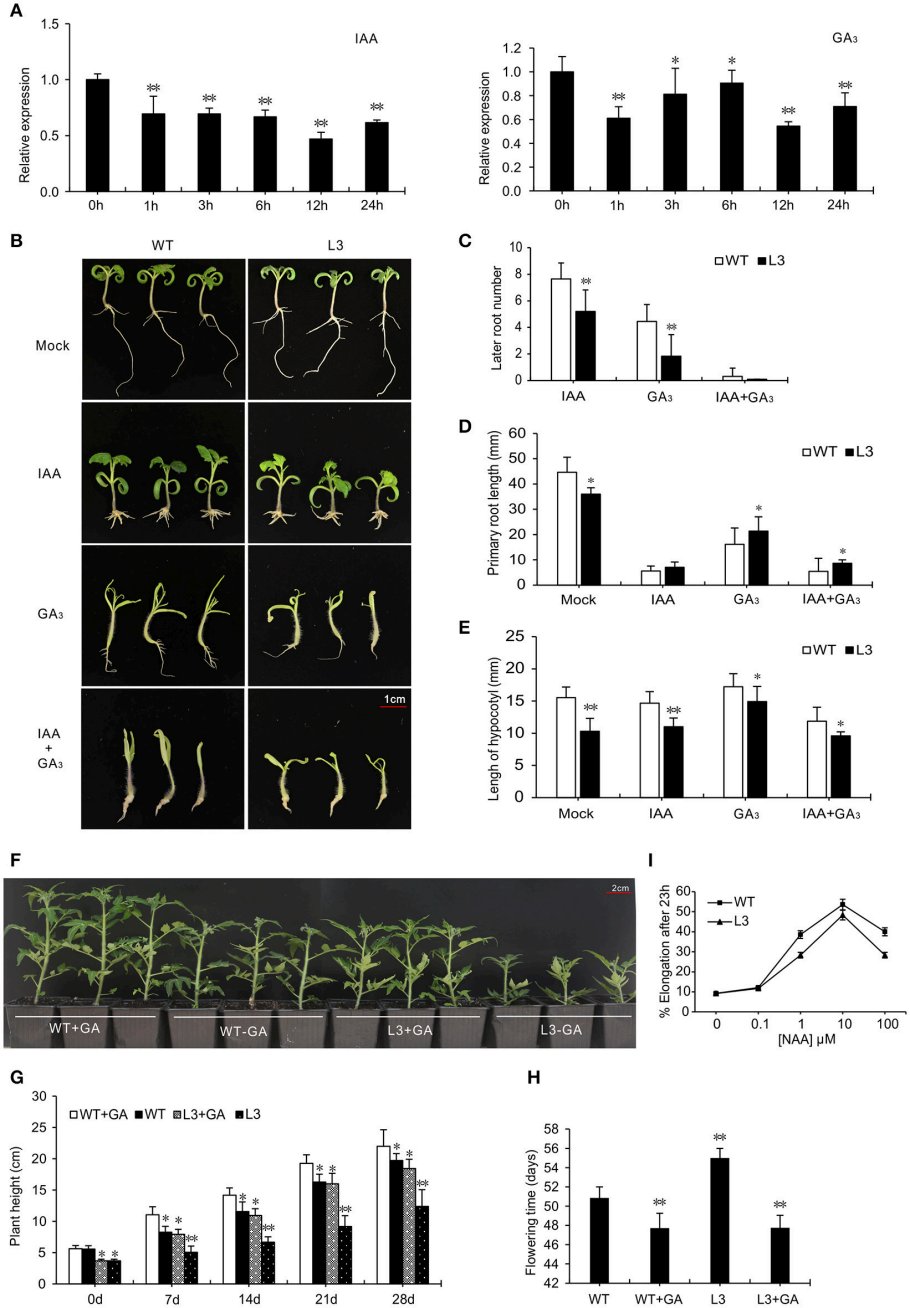
Overexpression of *SlGRAS40* alters responsiveness to IAA and GA_3_. **(A)** Quantitative RT-PCR analysis of *SlGRAS40* mRNA from leaves of 15-day-old WT seedlings treated with 20 μM IAA and 20 μM GA_3_. **(B)** Phenotypes of 15-day-old WT and *SlGRAS40*-OE L3 seedlings grown on ½ × MS medium containing 1 μM IAA and/or 50 μM GA_3_. **(C)** Number of lateral roots of WT and *SlGRAS40*-OE L3 seedlings treated with IAA, GA_3_ or IAA + GA_3_. **(D)** Primary root length of WT and *SlGRAS40*-OE L3 seedlings shown in **(B)**. **(E)** Hypocotyl length of WT and *SlGRAS40*-OE L3 seedlings shown in **(B)**. **(F)** Rescue of *SlGRAS40*-OE L3 dwarfism by exogenous GA_3_ application. **(G)** Plant height and **(H)** Phase transition time of GA_3_ treated plants shown in **(F)**. **(I)** Hypocotyl elongation of WT and *SlGRAS40*-OE L3 after NAA treatment. Asterisks indicate significant differences using Student's *t*-test (^*^P < 0.05, ^**^P < 0.01).

The expression level of *SlGRAS40* fell after IAA and GA_3_ treatment in WT (Figure 6A), indicating that *SlGRAS40* is responsive to auxin and gibberellin. To explore the relationship between *SlGRAS40* and the two phytohormones, WT and OE L3 seedlings were treated with 1 μM IAA or 50 μM GA_3_. *SlGRAS40*-OE seedlings had fewer lateral roots and longer primary roots than WT after IAA treatment (data of L3 in Figure 6B, data of L2 in Supplementary Figure S1), indicating that overexpression of *SlGRAS40* weakens the responsiveness to IAA. The auxin sensitivity of *SlGRAS40*-OE plants was explored by determining the auxin dose-response of elongation of hypocotyl segments. Maximum hypocotyl segment elongation was obtained with 10^−5^ M NAA both in OE L3 and WT, but the hypocotyl elongation of OE L3 was less than WT at each auxin concentration (Figure 6I). These data indicated that overexpression of *SlGRAS40* reduced hypocotyl auxin responsiveness.

In response to 50 μM GA_3_, hypocotyls of both WT and OE L3 seedlings elongated more than controls without GA_3_. Outgrowth of the first true leaves from the shoot apex was suppressed in OE L3 seedlings compared with WT, and WT seedlings had more lateral roots than OE L3 seedlings (Figure 6B, and data of L2 in Supplementary Figure S1). The dwarf phenotype and delayed flowering time could be rescued to a level similar to WT by spraying with 20 μM GA_3_ (Figure 6F). These results showed that overexpression of *SlGRAS40* induced GA-deficient phenotypes, so *SlGRAS40* may be involved in GA biosynthesis or signaling.

### Overexpression of *SlGRAS40* influences fruit size and disturbs fertilization

**Figure 7:**
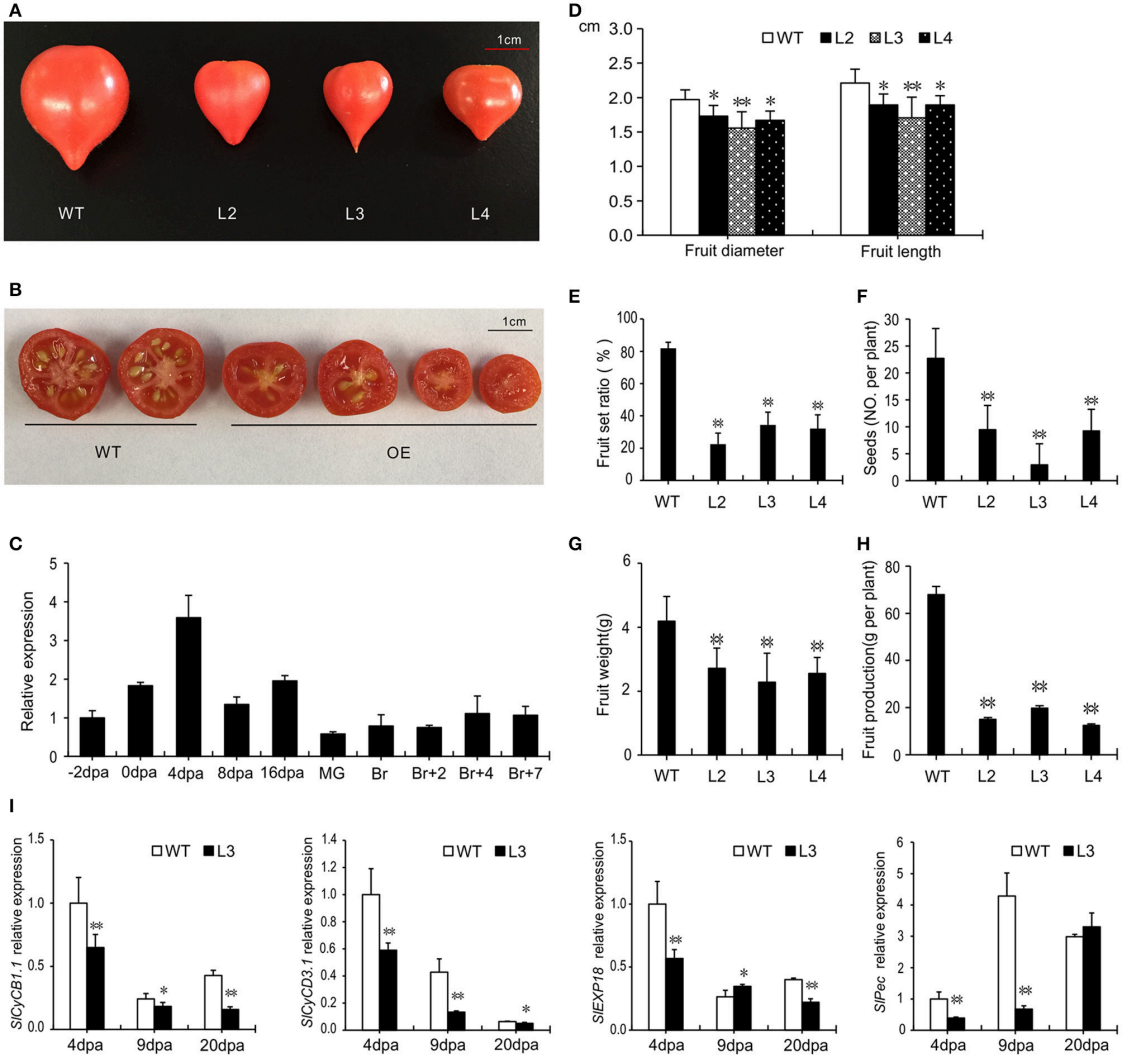
*SlGRAS40*-OE plants bear smaller fruits with fewer seeds. **(A,B)** Fruits of WT and *SlGRAS40*-OE plants. **(C)** Tissue profiling of *SlGRAS40* in different stage ovaries and fruits of wild-type tomato. dpa, days post anthesis; MG, mature green fruit; Br, color breaker fruit. The expression data was normalized with the value for −2 dpa ovary set to 1. **(D)** Diameter and length of WT and *SlGRAS40*-OE fruits in **(A)**. **(E)** Fruit set ratio, **(F)** Seed number, **(G)** Fruit weight, and **(H)** Fruit production of WT and *SlGRAS40*-OE fruits. Error bars show the standard error of values from three biological replicates (*n* = 3) with more than 20 fruits or 20 plants per replicate. **(I)** Quantitative RT-PCR analysis of cell division and expansion genes in 4, 9, and 20 dpa WT and *SlGRAS40*-OE L3 fruits. dpa, days post anthesis. Error bars show the standard error between three replicates. Asterisks indicate significant differences using Student's *t*-test (^*^P < 0.05, ^**^P < 0.01).

In plants overexpressing *SlGRAS40*, the fruit size was smaller than WT (Figures 7A,D). The normal fertilization process was also disrupted as the fruit set ratio and the number of seeds were significantly lower than in WT, and fresh weight and fruit production also decreased (Figures 7B,E–H). Studying the expression pattern at different stages of WT ovary and fruit development showed that *SlGRAS40* was up-regulated by pollination and fertilization, but has relatively low expression levels in fruits (Figure 7C). Final fruit size is controlled by genes related to cell division and cell expansion (Gillaspy et al., 1993), so we checked the expression levels of such genes in WT and OE L3 fruits harvested at 4, 9, and 20 dpa (Figure 7I). Genes related to cell division *SlCyCB1.1* (Solyc06g073610) and *SlCyCD3.1* (Solyc02g092980) were significantly down-regulated in 4 dpa, 9 dpa and 20 dpa OE fruits compared with WT. The gene *SlEXP18* (Solyc06g076220), which regulates cell expansion, was significantly down-regulated in 20 dpa OE fruits, and the gene *SlPec* (Solyc06g083580) was remarkably down-regulated in 4 dpa and 9 dpa OE fruit (Figure 7I).

**Figure 8:**
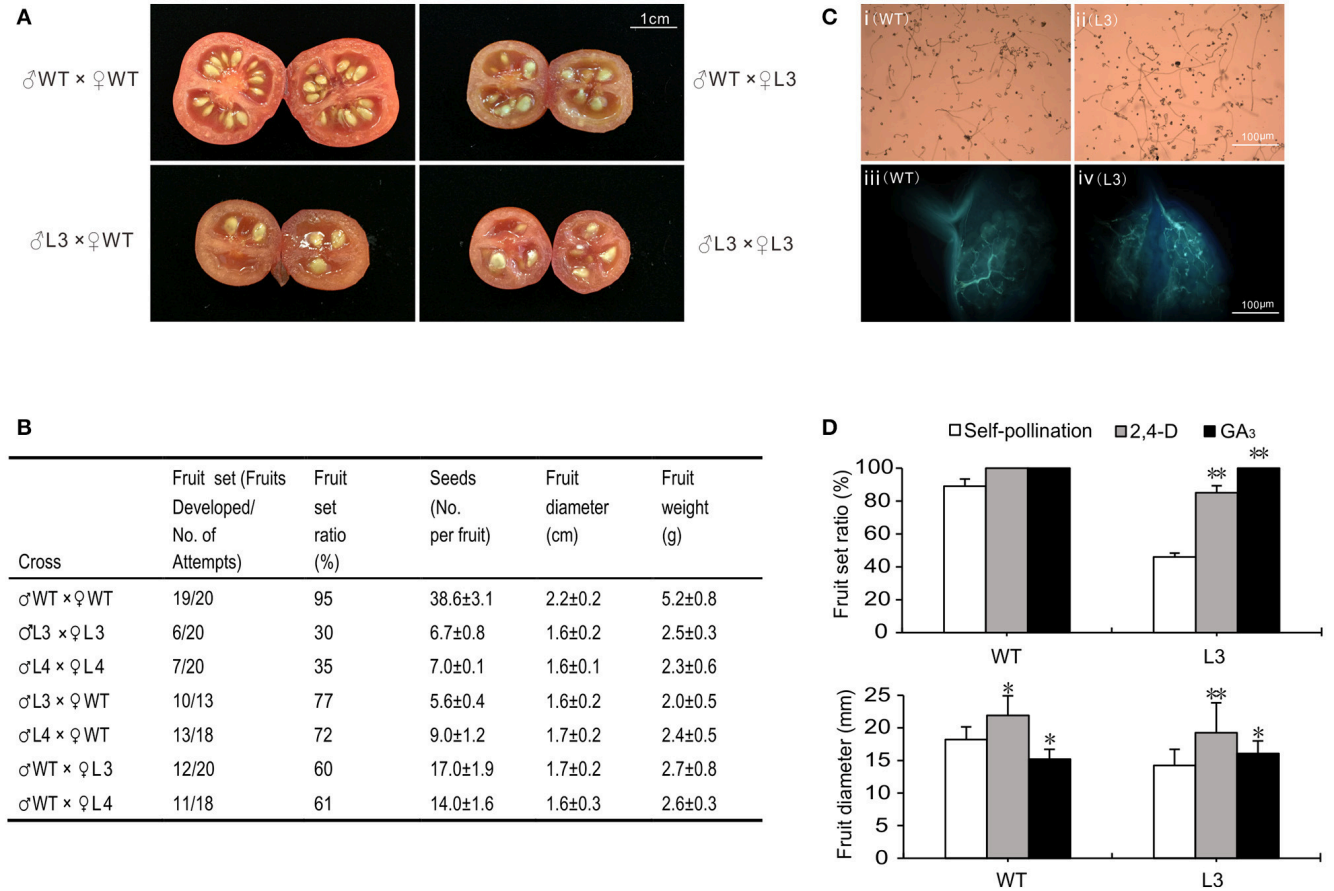
Overexpression of *SlGRAS40* disturbs fertilization. **(A)** Images of cross-fertilization assay. **(B)** Cross-fertilization assay data. **(C)** Pollen germination (i, ii) and pollen tube growth (iii, iv) of WT (i, iii) and *SlGRAS40*-OE L3 (ii, iv) plants. **(D)** Fruit-set ratio and fruit diameter of WT and *SlGRAS40*-OE L3 plants treated with 2,4-D and GA_3_ for 20 days. Error bars show the standard error between three biological replicates (*n* = 3) with more than eight ovaries per replicate. Asterisks indicate significant differences using Student's *t*-test (^*^P < 0.05, ^**^P < 0.01).

As the fruit-set ratio and seed number decreased, a cross-fertilization assay was performed to explore whether the stamens or pistils in *SlGRAS40*-OE plants were defective (Figure 8A). The fruit-set ratio was 95, 30, and 35% for WT, OE-L3 and OE-L4 self-pollination, respectively, and when WT was the female recipient the fruit-set ratio of WT fell to 77 and 72% with OE L3 and OE L4 pollen, respectively, while the seed number, fruit size and fresh weight all decreased. When OE L3 and OE L4 (♀) stigmas received WT pollen (♂), the fruit-set ratio increased to 60 and 61%, respectively, while the seed number, fruit size and fresh weight all increased slightly (Figure 8B). In pollen germination and pollen tube growth assays, the pollen from transgenic plants (OE L3) germinated normally and the pollen tube grew like WT. In OE L3 plants pollen tubes could grow through the stigma and style into the ovary, and then spread out toward the ovules as occurs in WT plants (Figure 8C). When emasculated ovaries (L3) were treated with 2,4-D or GA_3_ on the day equivalent to anthesis (0 dpa), fruit-set ratio and fruit size increased significantly (Figure 8D, Supplementary Figure S2), indicating that disruption of fertilization caused by *SlGRAS40* overexpression is related to altered auxin and gibberellin responses in *SlGRAS40*-OE pollinated ovaries.

### Differential expression of genes related to auxin and GA in *SlGRAS40*-OE plants

The expression of a range of genes related to auxin and GA was assessed in WT and OE L3 seedlings treated with IAA and GA_3_ for 3 h (Supplementary Figure S3). Expression of the following genes were tested: *SlIAAs, SlARFs, SlPINs, SlTIR1s, SlAFBs*, and *SlGH3*, all involved in auxin signaling; *SlGA20ox1, 3* and *4, SlGA3ox1* and *2*, and *SlGA2ox1, 2, 3, 4*, and *5*; genes encoding gibberellin receptors *SlGAST* and *SlGID1*; genes encoding gibberellin synthetases *SlKS, SlKAO, SlKO, SlCPS, SlGPS*; and *SlDELLA*, which is an inhibitor of gibberellin signaling. All these genes differed in how they were expressed in WT and OE seedlings. *SlPINs, SlTIR1s, SlAFBs, SlKO*, and *SlGPS* were all down-regulated in OE seedlings compared to WT. Also some genes responded differently after treatment with IAA or GA_3_ between OE and WT seedlings. *SlIAA4, SlIAA7, SlIAA9, SlARF5*, and *SlKS* were all up-regulated after GA_3_ treatment in WT, but were down-regulated in OE seedlings (Supplementary Figure S3). *SlARF6* was down-regulated in WT after IAA treatment, and up-regulated in OE seedlings. *SlAFB4* was down-regulated in WT after GA_3_ and IAA treatment, but up-regulated in OE seedlings. *SlDELLA* was up-regulated after GA_3_ and IAA treatment in WT, but down-regulated in OE seedlings after IAA treatment (Supplementary Figure S3). *SlGID1* and *SlGAST* were up-regulated after IAA treatment in WT, but down-regulated in OE seedlings compared with untreated seedlings. *SlGA2ox3* was strongly up-regulated in WT, but significantly down-regulated in OE after GA_3_ treatment. These results showed that overexpression of *SlGRAS40* altered the expression levels of genes involved in auxin and GA signaling, suggesting that *SlGRAS40* is involved in regulating the biosynthesis and/or signaling of auxin and GA in tomato.

### Expression levels of genes related to fruit set were altered in *SlGRAS40*-OE plants

In normal fruit development, the initiation of fruit set depends on the successful completion of pollination and fertilization, corresponding with a rise in levels of auxin and GA in pollinated ovaries (Serrani et al., 2007b). We compared gene expression of auxin and GA signaling genes related to fruit set in −2, 0, and 4 dpa ovaries, and ovaries treated with 2,4-D, or GA_3_ for 4 days (Supplementary Figure S4). All the auxin related genes have lower expression in 0 dpa OE ovaries compared with WT. *SlIAA9* was up-regulated in WT but down-regulated in OE ovaries after GA_3_ treatment. *SlGA20ox4, SlGA3ox1, 2*, and *SlGPS* had higher expression levels at −2 dpa that decreased rapidly at 0 dpa in OE ovaries compared with WT (Supplementary Figure S4). *SlGA2ox1, 3*, and *4* were all upregulated in −2 dpa OE ovaries, especially *SlGA2ox1*, which had a higher level in 0 dpa OE ovaries compared with WT (Supplementary Figure S4). *SlGID1* was expressed at a higher level in −2 dpa OE ovaries, but at a lower level at 0 dpa than in comparable WT fruit (Supplementary Figure S4). *SlDELLA* was upregulated in −2 and 0 dpa OE ovaries compared to WT. The disturbed fertilization in *SlGRAS40*-OE plants may have been due to the disruption in these auxin and gibberellin related genes.

### Overexpression of *SlGRAS40* modifies expression of genes involved in hormone signaling and stress responses in SAM

**Figure 9:**
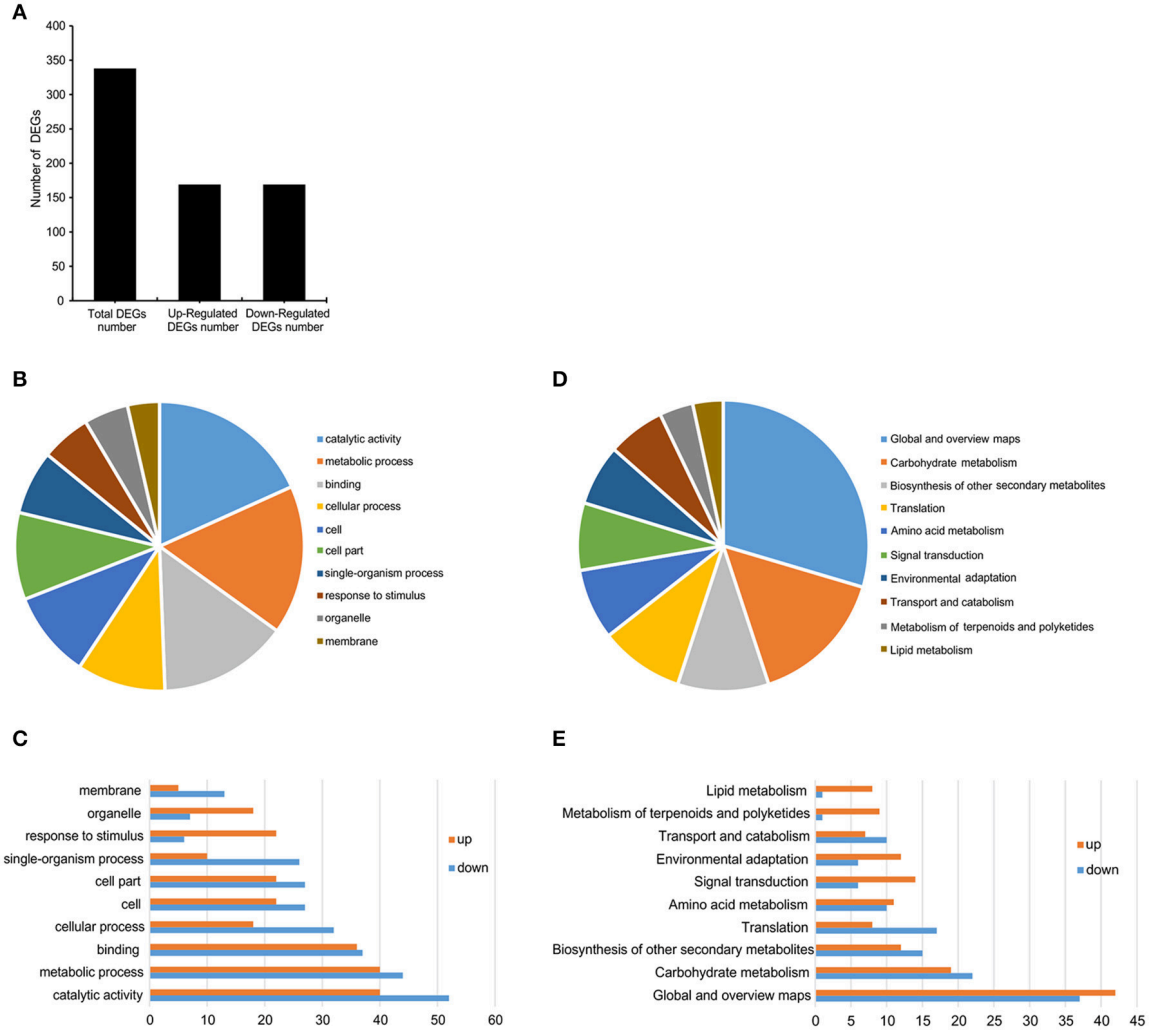
Classification of DEGs in RNA-Seq data. **(A)** The number of DEGs from the RNA-Seq data. **(B,C)** The top 10 GO terms of DEGs between *SlGRAS40***-**OE L3 and WT. **(D,E)** The top 10 KEGG pathways of DEGs between *SlGRAS40***-**OE L3 and WT.

To identify the changes in transcript levels of genes regulated by *SlGRAS40*, a comparative transcriptome analysis was conducted using shoot apical meristems (SAM) of 1-month-old *SlGRAS40*-OE L3 plants and WT controls. A total of 338 DEGs were identified, 169 upregulated and 169 downregulated (Figure 9A, Supplementary Table S3). A total of 30 GO terms (Supplementary Table S4) and 18 KEGG pathways (Supplementary Table S5) were enriched in the transcriptome of tomato overexpressing *SlGRAS40*. The top 10 GO terms (Figures 9B,C) and KEGG pathways (Figures 9D,E) were detailed in Figure 9. Overexpression of *SlGRAS40* influenced multiple processes including stress responses, phytohormone biosynthesis, signal transduction, transcription, primary and secondary metabolite biosynthesis, photosynthesis, and so on. There were 17 DEGs involved in hormone signaling including auxin, gibberellin, ethylene and abscisic acid, indicated hormones crosstalk may be influenced by *SlGRAS40*. And many transcription factors (TFs) were found in the data, including stress response transcription factors, such as NAC, WRKY, ERF, and MYB. Besides, such DEGs encoding positive regulators of stress resistance were induced by *SlGRAS40* overexpression, such as endochitinase, beta-amylase, polyphenol oxidase, zeaxanthin epoxidase and osmotin protein (Table 1), indicated the possible molecular mechanism that overexpression of *SlGRAS40* influences plant development and enhances abiotic resistance. A total of 9 genes were selected for supplementary qRT-PCR analysis (Supplementary Figure S5). For all the genes tested, qRT-PCR analysis validated the transcriptomic data. Sequencing clean data were uploaded to sequence read archive (SRA), the accession number was SRP115441.

**Table 1:** List of DEGs related to hormone signaling, transcription factor and stress response between WT and *SlGRAS40*-OE L3 tomato SAM (L3/WT, fold change ≥2.00 and probability ≥0.8).

**Gene ID**	**Functional annotation**	**log2 Fold**	**Probability**
**HORMONE SIGNALING**			
Solyc07g041720.1.1	Auxin-binding protein ABP19a-like	1.51	0.87
Solyc03g123410.1.1	Auxin-binding protein ABP19a	1.30	0.80
Solyc10g011660.2.1	Indole-3-acetic acid-amido synthetase GH3.5	1.43	0.84
Solyc10g008520.2.1	Auxin-responsive GH3-like	1.31	0.82
Solyc12g007230.1.1	IAA8 protein	−1.24	0.83
Solyc06g008590.2.1	IAA10 protein	−1.61	0.86
Solyc04g052910.1.1	SAUR family protein	−9.84	0.83
Solyc10g076680.1.1	Gibberellin 3-beta-dioxygenase	9.69	0.81
Solyc06g008870.2.1	Gibberellin receptor GID1B-like	1.44	0.81
Solyc06g069790.2.1	Gibberellin-regulated protein 6-like	1.27	0.83
Solyc02g083880.2.1	Gibberellin-regulated protein 11	1.32	0.84
Solyc03g116060.2.1	Gibberellin-regulated protein 6	−1.21	0.80
Solyc01g095140.2.1	Ethylene-responsive late embryogenesis-like protein	2.02	0.89
Solyc09g075420.2.1	Ethylene-binding protein	1.29	0.83
Solyc11g012980.1.1	Ethylene-responsive transcription factor ERF014	−3.48	0.82
Solyc01g095700.2.1	Abscisic acid receptor PYL8	1.45	0.85
Solyc03g095780.1.1	Abscisic acid receptor PYL4-like	1.30	0.81
**TRANSCRIPTION FACTORS**			
Solyc05g009310.2.1	Zinc finger protein CONSTANS-LIKE 16-like	1.99	0.83
Solyc01g111500.2.1	MYB-related protein 308-like	1.78	0.81
Solyc11g006720.1.1	Transcription factor DIVARICATA	1.76	0.83
Solyc03g034000.2.1	Transcription factor BEE 2	1.68	0.81
Solyc08g076820.2.1	Hop-interacting protein THI018	1.58	0.83
Solyc03g111710.2.1	BTB/POZ and TAZ domain-containing protein 1	1.48	0.84
Solyc01g086870.2.1	Transcription factor bHLH130-like	1.34	0.82
Solyc01g107190.2.1	LOB domain-containing protein 37-like	1.28	0.81
Solyc02g092930.1.1	Transcription factor MYB44-like	−1.25	0.82
Solyc04g015360.2.1	GATA transcription factor 8	−1.37	0.81
Solyc10g005080.2.1	MYB-related transcription factor LHY	−1.47	0.82
Solyc05g056620.1.1	MADS-box transcription factor MADS-MC	−1.63	0.82
Solyc11g012980.1.1	Ethylene-responsive transcription factor ERF014	−3.48	0.82
**STRESS RESPONSE (GENES WITH ASTERISKS MEANS TFS)**			
Solyc09g089520.2.1	Proteinase inhibitor I-B-like	8.11	0.98
Solyc09g084490.2.1	Wound-induced proteinase inhibitor 1-like	3.79	0.90
Solyc09g084480.2.1	Wound-induced proteinase inhibitor 1-like	2.46	0.90
Solyc09g083440.2.1	Wound-induced proteinase inhibitor 1	2.20	0.88
Solyc09g084470.2.1	Proteinase inhibitor 1	1.85	0.87
Solyc08g080660.1.1	Osmotin-like protein OSML15	2.68	0.86
Solyc10g074440.1.1	Endochitinase	2.15	0.89
Solyc10g055800.1.1	Endochitinase 4	1.10	0.81
Solyc08g077530.2.1	Beta-amylase 3	1.60	0.86
Solyc08g074620.1.1	Polyphenol oxidase E	1.57	0.87
Solyc08g074630.1.1	Polyphenol oxidase F	1.03	0.81
Solyc04g025650.2.1	Zeaxanthin epoxidase	1.06	0.80
Solyc05g052030.1.1^*^	Ethylene response transcription factor 4	2.46	0.88
Solyc05g052050.1.1^*^	Ethylene-responsive transcription factor 1	2.15	0.89
Solyc04g007000.1.1^*^	Transcription factor RAV1	1.90	0.87
Solyc04g071770.2.1^*^	Ethylene-responsive transcription factor ABR1-like	1.83	0.86
Solyc03g007410.2.1^*^	Transcription factor SPEECHLESS	1.80	0.82
Solyc06g060230.2.1^*^	NAC transcription factor	1.68	0.84
Solyc11g017470.1.1^*^	NAC transcription factor	1.44	0.83
Solyc02g088340.2.1^*^	WRKY transcription factor 3	3.71	0.91
Solyc03g116890.2.1^*^	WRKY transcription factor 40	1.20	0.85
Solyc07g008010.2.1^*^	Transcription factor MYB82	1.32	0.83
Solyc01g005440.2.1^*^	Jasmonate ZIM-domain protein 3	1.08	0.81
Solyc04g077980.1.1^*^	C2H2-type zinc finger transcription factor	−1.22	0.80
Solyc05g052570.2.1^*^	Zinc finger CCCH domain-containing protein 29-like	−2.52	0.90
Solyc03g026280.2.1^*^	Transcription factor CBF1	−4.27	0.87

### A hypothesized model of *SlGRAS40* enhances abiotic resistance interlinked with auxin and gibberellin

**Figure 10:**
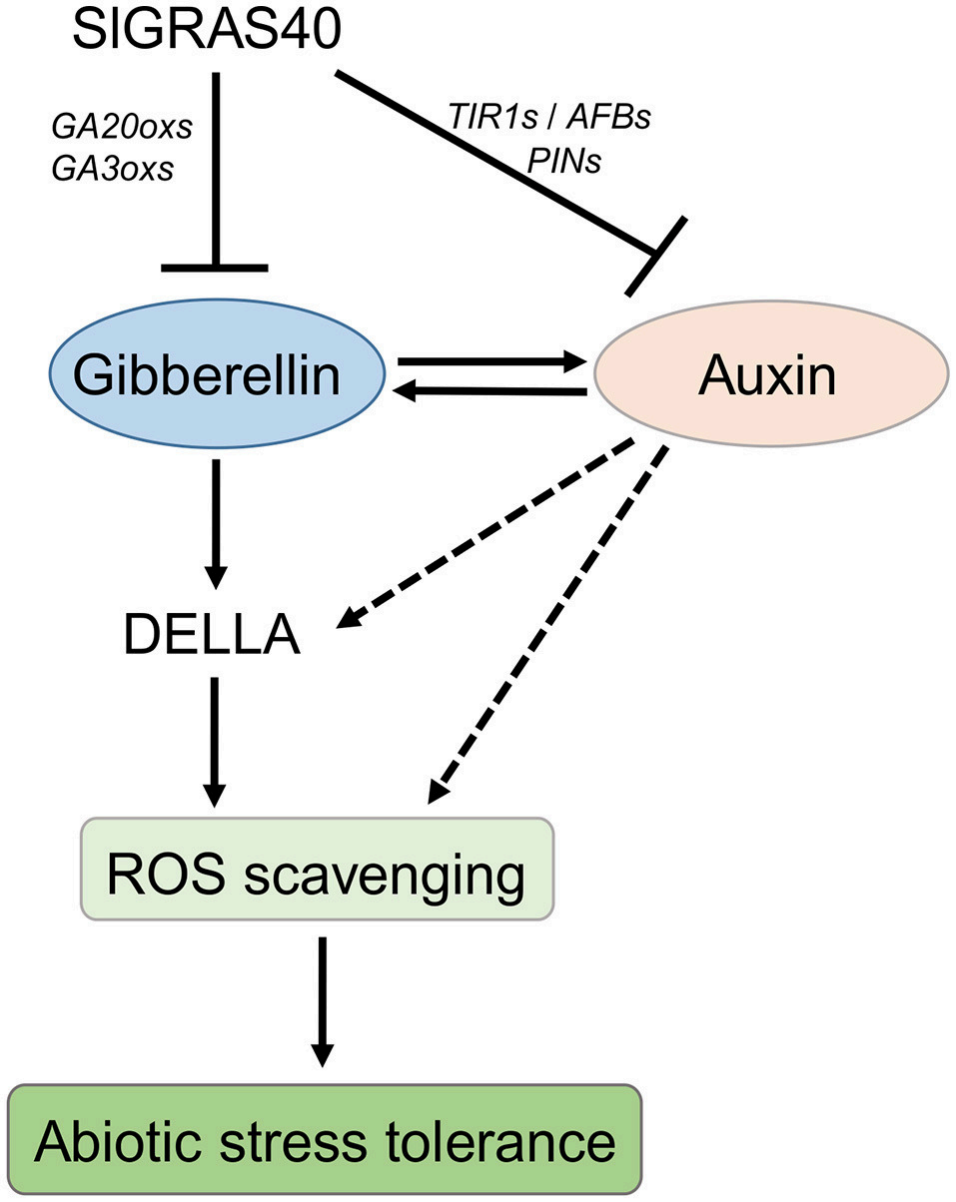
A hypothesized model of *SlGRAS40* enhances abiotic resistance interlink with auxin and gibberellin. *SlGRAS40* suppresses gibberellin biosynthesis and disrupts auxin signaling, and then influences auxin and gibberellin homeostasis. The crosstalk between auxin and gibberellin may be stimulate DELLA accumulation under abiotic stresses, and then enhances ROS scavenging ability and abiotic resistance.

*SlGRAS40* overexpressing plants displayed GA deficiency and auxin insensitivity (Figure 6). As GA biosynthesis-activating enzymes, *SlGA20ox1, SlGA20ox3, SlGA20ox4* and *SlGA3ox1, SlGA3ox2* were significant suppressed in *SlGRAS40*-OE plants (Supplementary Figure S2), may be induced lower bioactive GAs level in OE plants. And the expression of auxin receptors (*SlTIR1A, SlTIR1B, SlAFB4*, and *SlAFB6*) and auxin transporters (*SlPIN3* and *SlPIN6*) were also significant suppressed in *SlGRAS40*-OE plants (Supplementary Figure S2), suggested which impact auxin signaling in OE plants. These results indicated that overexpression of *SlGRAS40* in tomato disrupted auxin and gibberellin homeostasis and signaling. Accordingly, under abiotic stresses, we predicted that *SlGRAS40* influence auxin and gibberellin crosstalk, and enhance ROS scavenging ability mediated by DELLA stability in plant cells, and then empowered abiotic resistance in *SlGRAS40*-OE plants, and a proposed model was depicted in Figure 10.

## Discussion

### *SlGRAS40*-OE improved resistance to drought and salt via enhanced ability to scavenge ROS

Drought and salt stress can decrease photosynthetic capacity, augment oxidative damage to cells, and limit metabolic reactions (Farooq et al., 2009). In this work, damage symptoms in tomato plants, including wilting, chlorosis, and necrosis, were delayed in *SlGRAS40*-OE compared to WT under saline or drought stress (Figure 2B). Levels of RWC and total chlorophyll content were significantly higher in OE plants than in WT (Figures 2D–G). Seed germination rate and seedling root growth were less affected by D-mannitol and NaCl treatments in OE than in WT (Figure 5). These results suggested that overexpression of *SlGRAS40* enhanced the resistance to drought and salt stress during vegetative growth.

Abiotic and biotic stresses can trigger oxidative stress in plant cells (Dat et al., 2000) and the ROS generated may cause cell death if not adequately removed. The steady-state level of ROS within the cell represents a balance between the total reactive oxygen produced and the capacity of cellular antioxidant systems to remove it through both enzymatic and non-enzymatic means (Foyer and Noctor, 2005). In our research, H_2_O_2_ and ${\text{O}}_{2}^{-}$ contents were significantly lower in *SlGRAS40*-OE plants than in WT after drought and salt treatment (Figure 4A).

We hypothesized that *SlGRAS40*-OE plants had a better ROS scavenging ability. Ascorbate, glutathione, alkaloids, proline, and carbohydrates (Chen et al., 2007) act non-enzymatically as cellular redox buffers in plant cells (Apel and Hirt, 2004). *SlGRAS40*-OE plants accumulated more proline and soluble sugar than WT plants in response to both stresses. The MDA content increased significantly, a sign of oxidative damage, in WT plants under drought and salt stress conditions, but only a slight increase was observed in OE plants (Figure 4A). It is likely then that proline and soluble sugar protect *SlGRAS40*-OE against ROS, minimizing oxidative damage.

ROS scavenging enzymes in plants, including SOD, APX, CAT, POD, and GST, alleviate oxidative damage and enhance plant stress tolerance (Apel and Hirt, 2004). In this study, under normal and drought conditions, CAT activity was higher in OE plants than in WT (Figure 4A), and the activities of POD and SOD in *SlGRAS40*-OE plants were up-regulated under drought stress, but were unchanged in WT plants (Figure 4A). After salt treatment, activities of CAT, POD, and SOD were significantly up-regulated in WT plants, whereas OE plants had lower levels of these three activities (Figure 4A). There may be other antioxidant systems that play roles under salt stress in *SlGRAS40*-OE plants, but these data indicate that the ROS scavenging ability in *SlGRAS40*-OE plants was broadly enhanced.

Numerous genes have been reported to be upregulated under stress conditions in vegetative tissues (Seki et al., 2002; Zhu, 2002). In our research, the transcript levels of several genes involved in ROS scavenging were monitored (Figure 4B). Transcript levels of *SlchlAPX, SlCAT2, SlSOD, SlPOD*, and *SlLOX* plants were higher in *SlGRAS40*-OE than in WT both in control and stress treatments (Figure 4B). *SlGME2*, which encodes an important catalytic enzyme in ascorbic acid biosynthesis (Zhang C. J. et al., 2011), and *SlP5CS*, a key proline synthetase gene (Vendruscolo et al., 2007), accumulated more in OE plants than in WT both in normal and stress conditions (Figure 4B). *SlERF1*, an important regulator of abiotic/biotic stress responses (Lu et al., 2010), and the heat shock protein *SlHsp90-1* both had higher expression levels in OE plants after drought and salt stress treatment than in WT (Figure 4B). These results indicate that *SlGRAS40* modulates the expression of genes involved in stress signaling pathways, which might be a mechanism of enhancing the tolerance of drought and salt stress.

### Does *SlGRAS40* integrate phytohormone-regulated growth and stress responses in tomato?

Auxin and gibberellin have roles in abiotic stress responses in plants. For example, salt-activated ethylene and ABA signaling pathways integrate at the level of DELLAs to promote salt tolerance (Achard et al., 2006). DELLA proteins are also involved in ROS reactions and coordination of development during abiotic stress (Achard et al., 2008). Salt or mannitol treatment can enhance accumulation of DELLAs accompanied by upregulation of the genes encoding antioxidant systems, followed by a drop in ROS abundance (Apel and Hirt, 2004; Achard et al., 2006). A correlation has been made between endogenous auxin levels and abiotic stress responses in rice where overexpression of *OsPIN3t* or *OsGH3.13* increased tolerance to drought (Zhang et al., 2009, 2012). Previous studies also suggested that there is signaling reciprocity between auxin and ROS pathways. Exogenous application of auxin reduced the H_2_O_2_ content in tomato roots by increasing the expression and activity of H_2_O_2_ scavenging enzymes (Tyburski et al., 2009).

Some GRAS function as regulators of auxin and gibberellin in plant growth and development. In our previous study, we characterized how SlGRAS24 impacted multiple agronomical traits by regulating auxin and gibberellin homeostasis in tomato (Huang et al., 2017). In the present work, overexpression of *SlGRAS40* altered the responsiveness to IAA and GA_3_ (Figure 6B); led to auxin insensitivity and GA deficiency, and altered the abundance of transcripts related to auxin/gibberellin biosynthesis and signaling (Supplementary Figure S3). This is compelling evidence that *SlGRAS40* acts as a regulator of auxin and GA. We speculate that *SlGRAS40* regulates the response to abiotic stress in plant through auxin and/or GA signaling. A hypothesized model was shown in Figure 10, *SlGRAS40* suppresses gibberellin biosynthesis by decreasing the expression of genes encoding GA biosynthesis-activating enzymes, and disrupts auxin signaling by decreasing the expression of genes encoding auxin receptors and transporters, and then influences auxin and gibberellin homeostasis. Accordingly, we predict that the crosstalk between auxin and gibberellin may be stimulate DELLA accumulation under abiotic stresses in *SlGRAS40*-OE plants, and then enhances ROS scavenging ability and abiotic resistance. And there need more experiments to explore and make sure the network.

The comparative transcriptome analysis of *SlGRAS40*-OE L3 and WT plants showed that expression of a number of genes involved in auxin and gibberellin signaling were changed by *SlGRAS40* overexpression, besides some genes involved in ethylene and abscisic acid signaling also influenced (Table 1), indicated that the balance of phytohormones signaling crosstalk may be disrupted by overexpressing of *SlGRAS40* in tomato. On the other hand, the expression of many transcription factors were changed in *SlGRAS40*-OE plants (Table 1), may be reasons of pleiotropic phenotypes of *SlGRAS40*-OE plants. Endochitinase (Distefano et al., 2008), beta-amylase (Kaplan et al., 2006), polyphenol oxidase (Mahanil et al., 2008), zeaxanthin epoxidase (Zhang et al., 2016), and osmotin protein were known as positive regulators for stress resistance, these encoding genes were all significant induced by *SlGRAS40* overexpression, and many stress response transcription factors (Singh et al., 2002) including MYB, WRKY, NAC, and ERF were also induced expressing in *SlGRAS40*-OE plants (Table 1). Among these changes could be the way in which *SlGRAS40* influences plant development and resistance to abiotic stress.

We also found that overexpression of *SlGRAS40* impacted fruit-set ratio and fruit development (Figure 7), including expression of genes related to auxin and gibberellin signaling during fruit set (Supplementary Figure S4). Caution should be taken when applying auxin and gibberellin to tomato crops, because these hormones not only modulate stress responses, but also influence plant growth and development, fertilization and fruit development.

Taking together, our study of *SlGRAS40* provides evidence that another GRAS potentially plays an integrating role in tomato, regulating resistance to abiotic stresses and auxin and gibberellin signaling during vegetative and reproductive growth.

## Author contributions

ZL, YL, and WH designed the experiments; YL, NH, HR, JC, and DS performed the experiments; YL and DL analyzed the data; YL and WH wrote the manuscript; ZL and ZX revised the manuscript.

### Conflict of interest statement

The authors declare that the research was conducted in the absence of any commercial or financial relationships that could be construed as a potential conflict of interest.
