# Supplementary material for: Overexpression of SlGRAS40 in Tomato Enhances Tolerance to Abiotic Stresses and Influences Auxin and Gibberellin Signaling
Source: Front Plant Sci. 2017 Sep 26;8:1659. doi: 10.3389/fpls.2017.01659 (PMC5622987; doi:10.3389/fpls.2017.01659)
Supplement: Supplementary file 1 [file Table1.DOCX]

**Supplementary Table S1.** Primers used for qRT-PCR

|  | Primers for qRT-PCR |  |
| --- | --- | --- |
| Gene Name | Forward (5’-3’) | Reverse (5’-3’) |
| *SlGRAS40* (Solyc08g078800) | TTGGAGGAGCTCTATTGCCT | CTTTGGCTGAAACTGGTGAG |
| *SlIAA3* (Solyc09g065850) | CTCAGGAATGTATTTAAAAGTTAG | TCCTTCTCTTTCTGAATACACT |
| *SlIAA4* (Solyc06g053840) | AACAAGAGGGCTTTGCCTGAG | GTGTCTTGGCAACAGGTGGA |
| *SlIAA7* (Solyc06g053830) | ACTCAACCTCCATCATAATGATAATATTCC | ACCCCACCACTTGAGCCTTA |
| *SlIAA8* (Solyc03g120390) | CTTGCCTAACAATCTGTAATTC | TGTTCTTGGAGCTAATCCTATA |
| *SlIAA9* (Solyc04g076850) | CCCCTTGCACCCTTCCA | AGCGTCTGAAAATCCTCGTTTG |
| *SlARF5* (Solyc04g081240) | ATTAGTTCTGAGTTGTGGC | GGTATCTGTGAAGTTGCTG |
| *SlARF6* (Solyc07g043460) | GGTTCAACGGGTTCTCAAC | TTCAGGGAAGTGGATGCTC |
| *SlARF7* (Solyc07g042260) | CCAAGTTATCCTAATCTTCCTTCC | GTAAAGCCTCCTGGTCATATTTG |
| *SlARF8* (Solyc02g037530) | CTGCTCAAACCCAAATGCTGTC | GGTAAGTGTGTTGGTGAGCCTG |
| *SlPIN1* (Solyc03g118740) | GCTGCAGGCTGGTCTAGATT | AACAATGGCAACAAAGCACA |
| *SlPIN3* (Solyc04g007690) | TTCAAAATCAATTTAGCGTGTCA | CTCAAAATCCCTCTTGTTTCG |
| *SlPIN5* (Solyc01g068410) | ACATTGAGCTGGCATTTTGG | TCCACTACCAGCCTTTGACA |
| *SlPIN6* (Solyc06g059730) | AGATGGCAGCAATAGGGATG | GCGAAGACAAATGGAACGAT |
| *SlTIR1A* (Solyc09g074520) | GGTGATAGCGATCTGGGACT | CTCGAGCTTTGCAGCATTAG |
| *SlTIR1B* (Solyc06g008780) | GGCTCTGTTGGCTATTGCTGC | CATTGCTATATGGAGTCGATG |
| *SlAFB4* (Solyc04g074980) | ACGGTCACTGATGAACCAGA | TGCAGGCAGATATTCAGGAG |
| *SlAFB6* (Solyc02g079190) | GAATCAATGCGGTGTCTTTG | GTCATCGCTGTGCTCATCTT |
| *SlGH3* (Solyc07g053030) | TGTGACATAGTCCCAGTAACAATAACATCG | TTGAAATGGAATGTAGTAAAGAGTCATGGAAAGG |
| *SlGPS* (Solyc08g023470) | TATGCAGAAAACATATTACAAGA | ATCAAGAACATCATCTATTAATTG |
| *SlCPS* (Solyc06g084240) | ATACCTAGAGCTAGCGAAATC | ACTGCCTAAATAGTACGTAACC |
| *SlKS* (Solyc07g066670) | TGATTTCTTTGATGTAGGAGGTTC | GCTTGCCACTTAGATGCTTTG |
| *SlKO*（Solyc04g083160） | CCACGAAGACACGCAGGTAG | ATCGTTCAGGCTTCCACTCTT |
| *SlKAO*（Solyc01g080900） | CTTTCAAATCCAACAATCCTG | TTAAAACCTTCCTGCAACCT |
| *SlGA20ox1*（Solyc03g006880） | CTCATTTCTAATGCTCATCGT | TGCAGATGATTCTTTCTTAGCG |
| *SlGA20ox2*（Solyc06g035530） | TTTCCATATTCTACCCTACAAG | TCATCGCATTACAATACTCTT |
| *SlGA20ox3*（Solyc11g072310） | AGCCAAATTATGCTAGTGTTAC | TTTTATGAGATTTGTGTCAACC |
| *SlGA20ox4*（Solyc01g093980） | GATGATAAATGGCACTCTATTC | TGACTTCCTTGTTCTTCTACAG |
| *SlGA3ox1*（Solyc06g066820） | GGCATTAGTAGTTAATATAGGTGA | AAATAAGCTACAGAAAGTCGATA |
| *SlGA3ox2*（Solyc03g119910） | GATCATAAATTTGTCATGGATAC | TGTTTCCATATGGTTAAGTAATC |
| *SlGA2ox1*（Solyc05g053340） | GGCATGTAAGATATTAGAATTGA | TTAATCCGTAGTAGAGAATCAGA |
| *SlGA2ox2*（Solyc07g056670） | ATTAAGATCCAATAACACTTCG | TCTTGATTTCACACTATTTGC |
| *SlGA2ox3*（Solyc01g079200） | GACCCTTCTACTTTCAGCTC | AAATTGAATTGTCTTCTATCCA |
| *SlGA2ox4*（Solyc07g061720） | ATGGAAGGAAAAGACAGTTTA | CTTTTCTCAAATAGGACCAAC |
| *SlGA2ox5*（Solyc07g061730） | GATCACTTACCAATAATCAACAG | CGTCATGGTTTACGACTTTA |
| *SlGAST1*（Solyc02g089350） | CAACAACAGAGAAATAACCAAC | TTATACGATGTCTTTGAACACC |
| *SlDELLA*（Solyc11g011260） | TGATGCGACTATACTTGATATAAG | GGGTTAATCTGTTTAATAGAGTTC |
| *SlGID1*（Solyc09g074270） | GATCTTGATACACCTCTCAGTACTA | ACAGCCTTACATATACTAACAAGAC |
| *SlCycB1.1* (Solyc06g073610) | CGTTACTAGGAGGTCTGCTG | CCTTTAGTTACAAGAGGCTTCG |
| *SlCycD3.1* (Solyc02g092980) | TTTAGATTCCCAGCTCCAAAATCCTA | TTCATTTCCGACATCTAAACTAGACC |
| *SlEXP18* (Solyc06g076220) | ATTGGATAAGTATGAGCAGGAACTGG | ATGAAGTAGAGCTCCTTCTATCACC |
| *SlPec* (Solyc06g083580) | ATGGGAAGGATCATGGAGACAGTGG | AAGGAAGAGGACTTCGCAGCTAAGC |
| *SlUBI* (Solyc07g064130) | GCCGACTACAACATCCAGAAGG | TGCAACACAGCGAGCTTAACC |
| *SlCAT* (Solyc12g094620) | AAGTCCTGTGGTCAGAAGGTCG | GAAGTACAGTTTATAGCACAACGCG |
| *SlSOD* (Solyc01g067740) | TGAATTGGGGTTGAACCATT | GCAGGCACTGTAATCTGCAA |
| *SlPOD*（Solyc11g018800） | CTTGCCCTAATGCTCTCACC | GCATCACAACCCTGAACAAA |
| *SlGST* (Solyc09g011590) | GCAAGCCCATTTGTGAGTCT | TGCTGACCCCTTATCATCG |
| *SlLOX* (Solyc01g006560) | TCATTTTCCCCTGGCAAGTA | TGGTGCATTTGGATCTTCCT |
| *SlAPX* (Solyc06g005160) | ACGATGATATTGTGACACTCTTCCA | AAGCGATGAAACCACAAAAACA |
| *SlP5CS* (Solyc08g043170) | TGCTGTAGGTGTTGGTCGTCA | TGCCATCAAGCTCAGTTTGTG |
| *SlERF1* (Solyc03g093610) | TTTTAGTATCGGATGGACG | GGCGGAGAAACAGAAGTA |
| *SlHsp90-1* (Solyc12g015880) | GCACTTCTCTGTTGAAGGTCAG | ATGAACACACGGCGAACATA |
| *SlGEM2* (Solyc09g082990) | CCATCACATTCCAGGACCAGA | CGTAATCCTCAACCCATCCTTC |
| *SlER5* (Solyc01g095140) | TATTGGTAAAGATTGGGACATTGA | TGTCTTCTTGTTTGTCACCGTTC |
| *Solyc12g007230.1.1* | ACTTAGACTAGGCTTGCCTG | AACTTTTACACCCTTTGGAG |
| *Solyc03g095780.1.1* | ACGCTGCATCCGGCGGATGA | CAGCGATTTGCAGTTGCGAT |
| *Solyc05g052050.1.1* | CGGTGTATCGCCGGAATT | CCATCCGACGCTTAGAGC |
| *Solyc08g077530.2.1* | GCAGTATTCCTCTACCTC | TCGGGATTTCTCCGGCCTG |
| *Solyc08g074620.1.1* | ACCCGATGATATCGATAGT | AGGGTCAAAGGGGTCTTTA |
| *Solyc04g025650.2.1* | ATCAGAGGGAGCAGCCATAG | CCATGTAATTCGCATCCCTT |
| *Solyc04g071770.2.1* | TATTAAAGGTGGCGAATCA | TGACTATACCCTGTATACA |
| *Solyc01g005440.2.1* | TTACTTGTAGTCAAGGAT | AGGCTGACGTTTGAGACC |
| *Solyc03g007410.2.1* | TATTCGACGATTCCGAA | ATACCACTATTACCACC |
