# Supplementary material for: Overexpression of SlGRAS40 in Tomato Enhances Tolerance to Abiotic Stresses and Influences Auxin and Gibberellin Signaling
Source: Front Plant Sci. 2017 Sep 26;8:1659. doi: 10.3389/fpls.2017.01659 (PMC5622987; doi:10.3389/fpls.2017.01659)
Supplement: Supplementary file 2 [file Table2.DOCX]

**Supplementary Table S2.** Phenotypes of WT and *SlGRAS40*-OE plants.

| Parameter | WT | L2 | L3 | L4 |
| --- | --- | --- | --- | --- |
| Plant height (one month old, cm) | 17.4±0.47 | 7.8±1.9** | 7.2±1.9** | 10.3±2.9** |
| Plant height (two months old, cm) | 29.7±2.0 | 20.0±1.6** | 19.6±3.0** | 21±1.4** |
| Plant height (three months old, cm) | 31.5±2.4 | 21.2±3.1** | 11.5±3.2** | 20.2±3.2** |
| Stem length of sixth internode (two months old, cm) | 2.1±0.2 | 1.2±0.2** | 1.2±0.3** | 1.3±0.2** |
| Stem diameter of sixth internode (two months old, cm) | 0.6±0.04 | 0.4±0.05* | 0.4±0.04* | 0.4±0.04* |
| Leave length of sixth node (two months old, cm) | 5.3±0.3 | 3.9±0.3* | 3.7±0.4* | 3.9±0.8* |
| Leave width of sixth node (two months old, cm) | 2.4±0.1 | 1.7±0.1* | 1.8±0.1* | 1.5±0.1* |
| Days to first visible flower bud | 30.8±1.2 | 44.2±3.7** | 44.6±2.5** | 49±2.8** |
| Days to anthesis of first flower | 46.6±4.0 | 59.5±2.6** | 59.1±2.9** | 63.5±2.1** |
| Leaves to first inflorescence (n) | 9.7±1.1 | 7.5±0.9* | 7.1±1.3* | 8.6±1.0* |

Note: Values are means of at least 25 plants, ± SE. The statistical significance of mean differences was analysed using a *t*-test: *P < 0.05, **P < 0.01.
