# Supplementary material for: Overexpression of SlGRAS40 in Tomato Enhances Tolerance to Abiotic Stresses and Influences Auxin and Gibberellin Signaling
Source: Front Plant Sci. 2017 Sep 26;8:1659. doi: 10.3389/fpls.2017.01659 (PMC5622987; doi:10.3389/fpls.2017.01659)
Supplement: Supplementary file 6 [file Presentation1.PDF]

Supplementary Material

Overexpression of *SlGRAS40* in tomato enhances tolerance to abiotic stresses and influences auxin and gibberellin signaling

Yudong Liu, Wei Huang, Zhiqiang Xian, Nan Hu, Dongbo Lin, Hua Ren, Jingxuan Chen, Deding Su, Zhengguo Li*

School of Life Sciences, Chongqing University, Chongqing, China

*** Correspondence:**
Zhengguo Li
Zhengguoli@cqu.edu.cn

Supplementary Table S1. Primers used for qRT-PCR.

Supplementary Table S2. Phenotypes of WT and *SlGRAS40*-OE plants.

Supplementary Table S3. The total gene transcripts and DEGs detected in tomato overexpressing *SlGRAS40*.

Supplementary Table S4. The enriched GO terms in tomato overexpressing *SlGRAS40*.

Supplementary Table S5. The enriched KEGG pathways in tomato overexpressing *SlGRAS40*.

Supplementary Figure S1. Overexpression of *SlGRAS40* alters responsiveness to IAA and GA_3 in line L2.
(A) Phenotypes of 15-day-old WT and *SlGRAS40*-OE L2 seedlings grown on ½ × MS medium containing 1μM IAA and/or 50μM GA_3.
(B) Number of lateral roots of WT and *SlGRAS40*-OE L2 seedlings treated with IAA, GA_3 or IAA + GA_3.
(C) Primary root length of WT and *SlGRAS40*-OE L2 seedlings shown in **(A)**.
(D) Hypocotyl length of WT and *SlGRAS40*-OE L2 seedlings shown in **(A)**.

Supplementary Figure S2. Fruits of WT and *SlGRAS40*-OE L3 plants treated with 2,4-D and GA_3 for 20 days.

Supplementary Figure S3. Expression analysis of GA/auxin-related genes after

GA_3/IAA treatment.
Quantitative RT-PCR analysis of GA- and auxin-related genes in 15-day-old WT and *SlGRAS40*-OE L3 seedlings and in response to GA_3 or IAA treatment (20 μM for 3 h). Error bars show the standard error of values from three replicates. Asterisks indicate significant differences using Student's *t*-test (*P < 0.05, **P < 0.01).

Supplementary Figure S4. Expression analysis of GA/auxin-related genes during fruit set.
(A) and **(B)** Quantitative RT-PCR analysis of GA/auxin-related genes in WT and *SlGRAS40*-OE L3 ovaries. dpa, days post anthesis. Error bars show the standard error of values from three replicates. Asterisks indicate significant differences using Student's *t*-test (*P < 0.05, **P < 0.01).

Supplementary Figure S5. Quantitative RT-PCR validation of transcriptomic data.
Three hormone signaling-related genes, three stress-related genes and three transcription factor genes were selected and their expression validated by qRT-PCR. Error bars show the standard error of values from three biological replicates (n = 3).
