# Supplementary figures and images for: Overexpression of SlGRAS40 in Tomato Enhances Tolerance to Abiotic Stresses and Influences Auxin and Gibberellin Signaling
Source: Front Plant Sci. 2017 Sep 26;8:1659. doi: 10.3389/fpls.2017.01659 (PMC5622987; doi:10.3389/fpls.2017.01659)

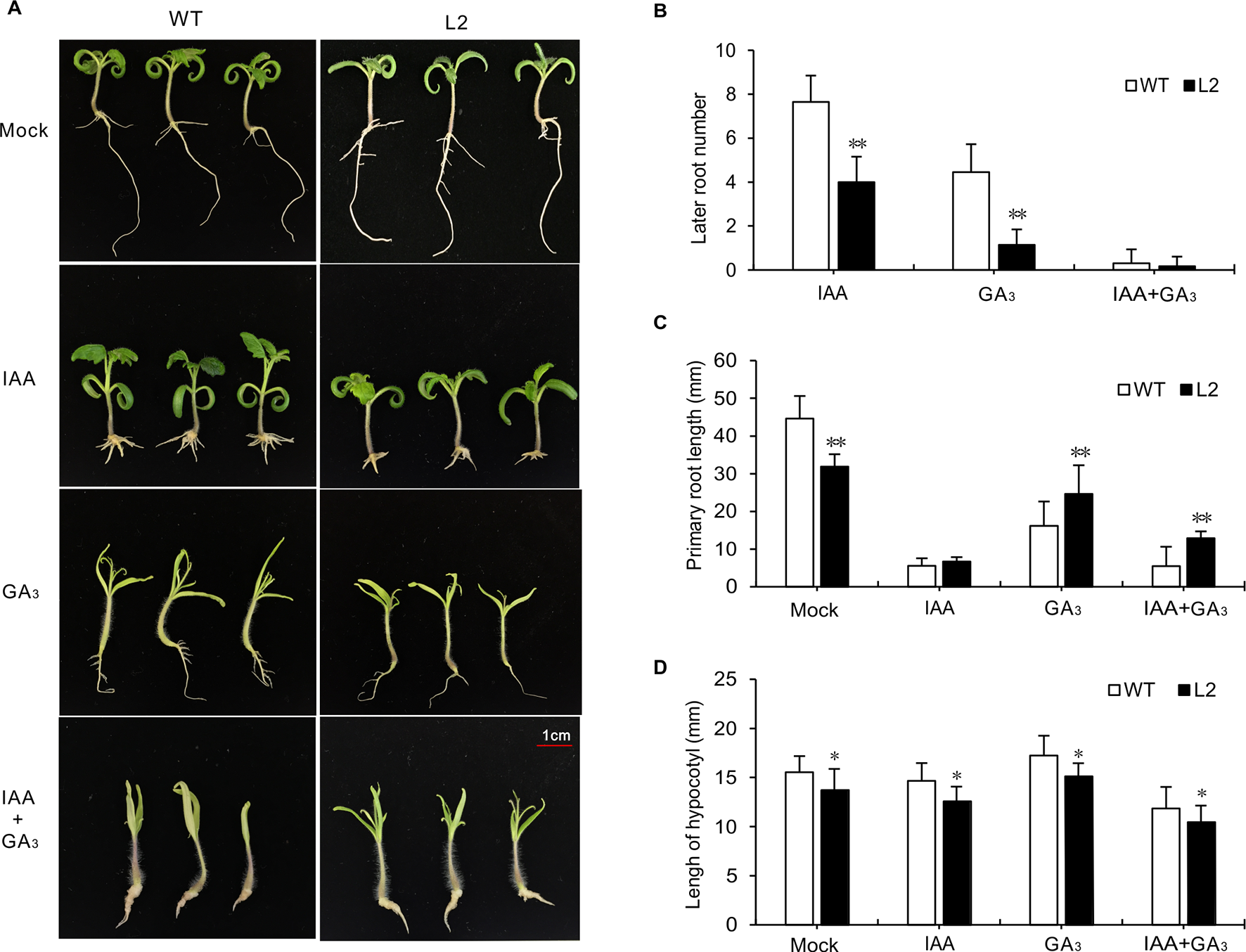

Supplement: Supplementary file 7 [file Image1.TIF]

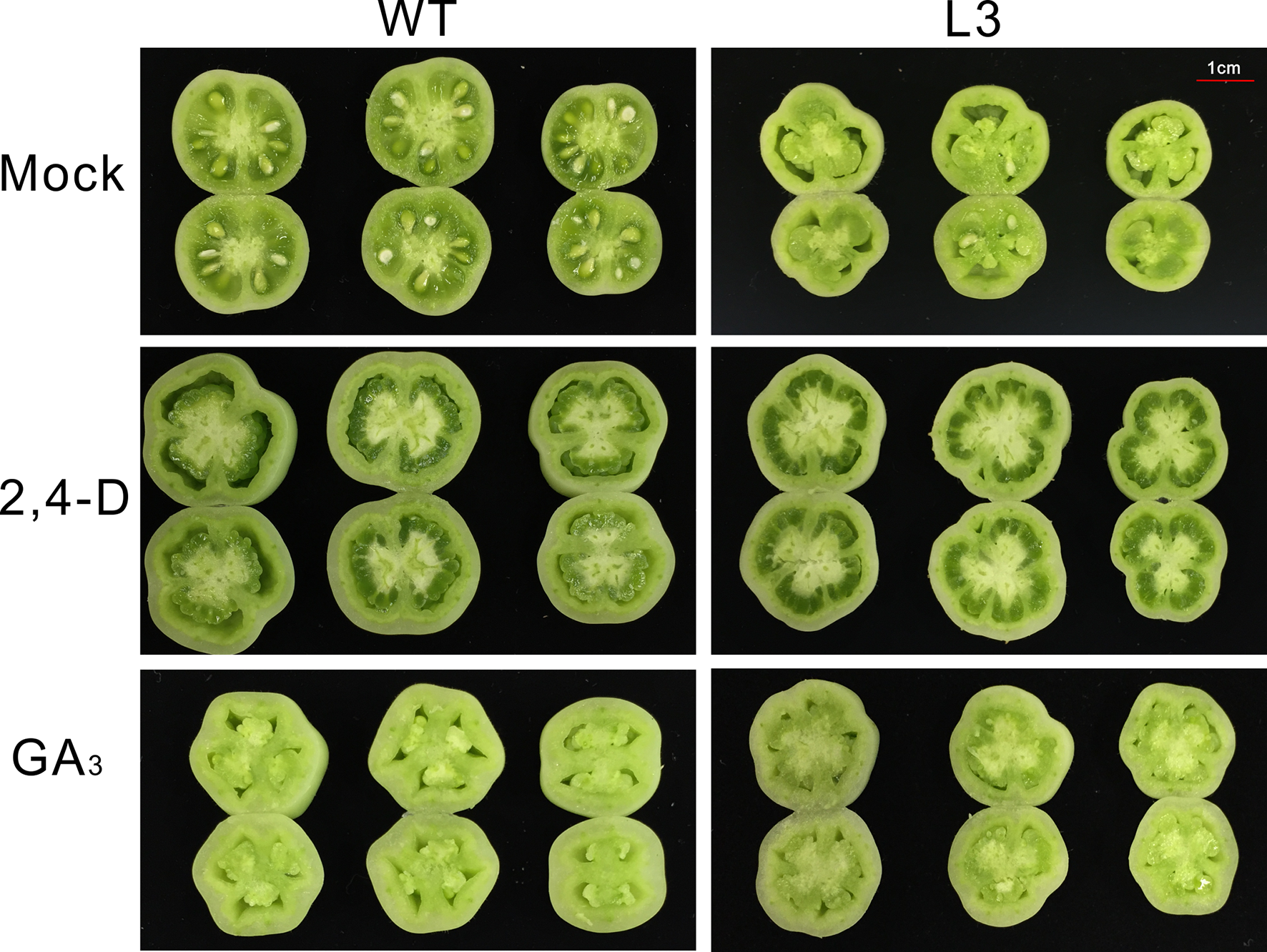

Supplement: Supplementary file 8 [file Image2.TIF]

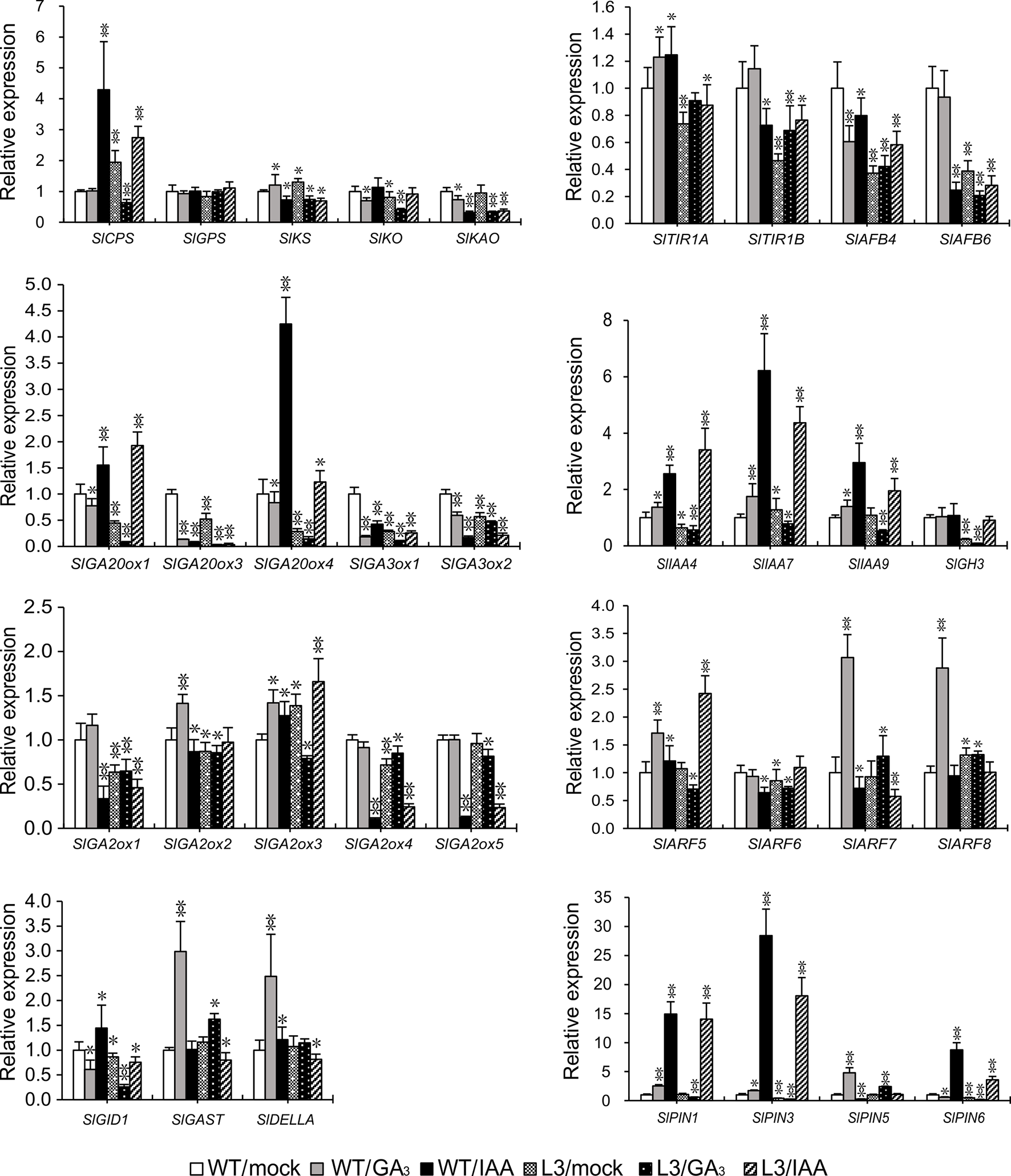

Supplement: Supplementary file 9 [file Image3.TIF]

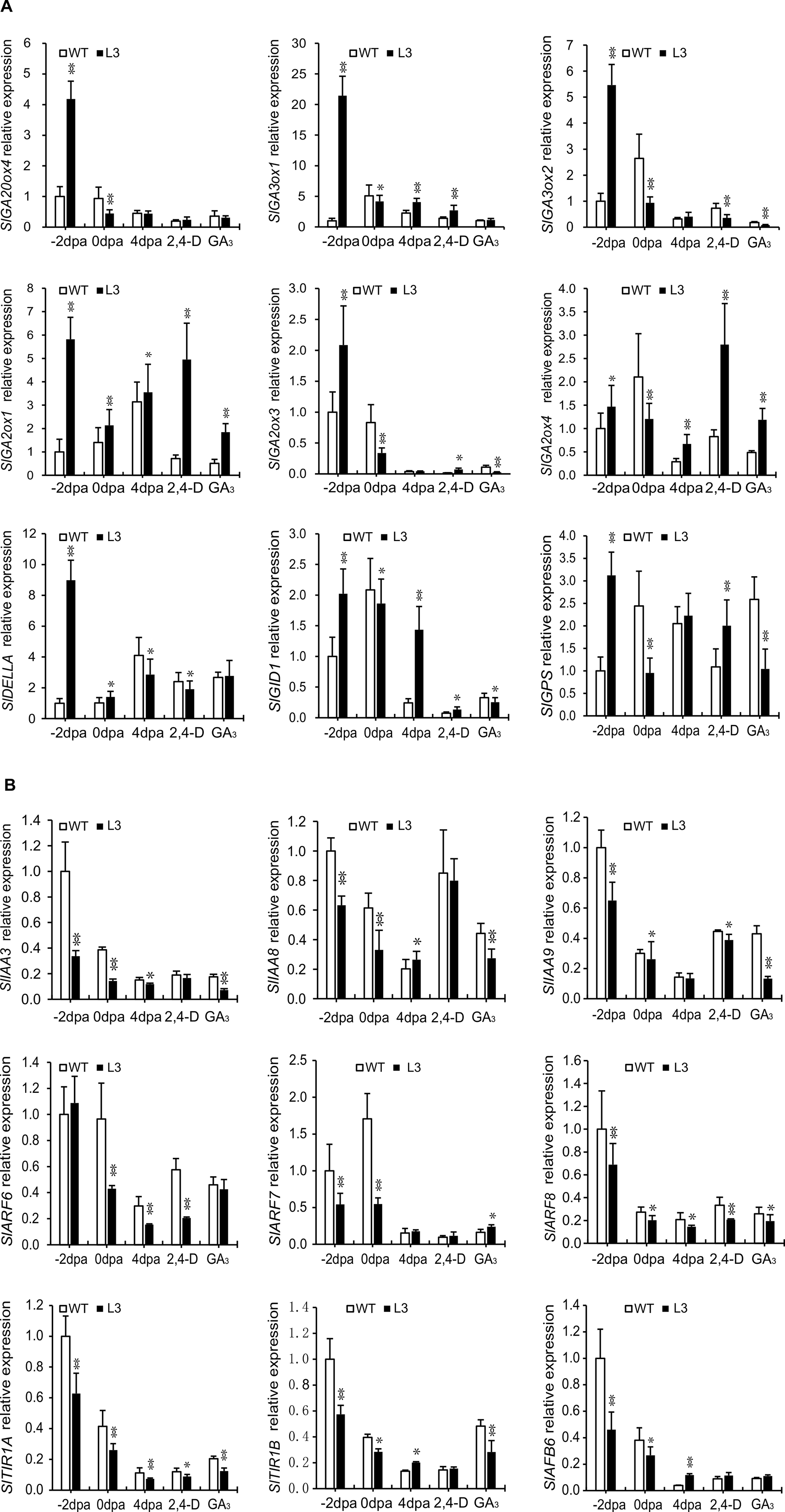

Supplement: Supplementary file 10 [file Image4.TIF]

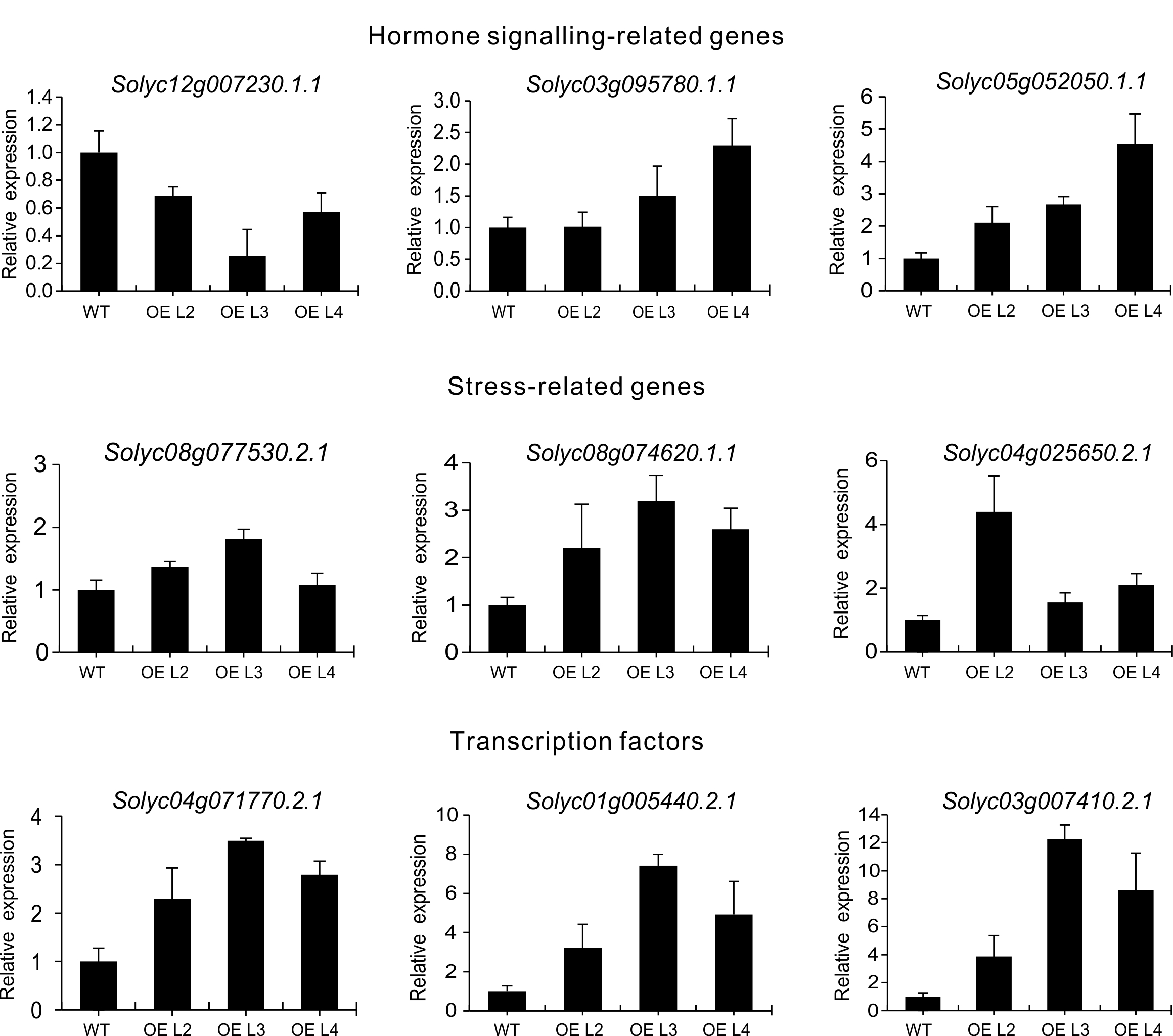

Supplement: Supplementary file 11 [file Image5.JPEG]
